# Pan-tumor landscape of *fibroblast growth factor receptor 1-4* genomic alterations*

**DOI:** 10.1016/j.esmoop.2022.100641

**Published:** 2022-11-30

**Authors:** K. Murugesan, A. Necchi, T.C. Burn, O. Gjoerup, R. Greenstein, M. Krook, J.A. López, M. Montesion, H. Nimeiri, A.R. Parikh, S. Roychowdhury, S. Schwemmers, I.M. Silverman, A. Vogel

**Affiliations:** 1 Cancer Genomics Research, Foundation Medicine, Inc., Cambridge, USA; 2 Genitourinary Medical Oncology, Vita-Salute San Raffaele University, Milan; 3 Genitourinary Medical Oncology, IRCCS San Raffaele Hospital and Scientific Institute, Milan, Italy; 4 Translational and Data Sciences, Incyte Corporation, Wilmington; 5 Scientific and Medical Publications, Foundation Medicine, Inc., Cambridge, USA; 6 Research Scientist, Ohio State University, Columbus, USA; 7 Integrated Healthcare Solutions, F. Hoffmann-La Roche Ltd, Basel, Switzerland; 8 Global Clinical Development Lead Oncology, Foundation Medicine, Inc., Cambridge, USA; 9 Oncology (Medical/Hematology), Jefferson Health, Philadelphia, USA; 10 Medical Oncology, Ohio State University, Columbus, USA; 11 Integrated HealthCare Solutions PDMA (Oncology), F. Hoffmann-La Roche Ltd, Basel, Switzerland; 12 Clinical Bioinformatics, Incyte Corporation, Wilmington; 13 Clinic for Gastroenterology, Hepatology & Endocrinology, Hannover Medical School, Hannover, Germany

**Keywords:** *FGFR1-4*, FGFR inhibitors, gene rearrangements/fusions, short variants, targeted therapy

## Abstract

**Background:**

Selective tyrosine kinase inhibitors targeting *fibroblast growth factor receptor* (*FGFR*) *1-4* genomic alterations are in development or have been approved for *FGFR*-altered cancers (e.g. bladder cancer and advanced intrahepatic cholangiocarcinoma). Understanding FGFR inhibitor-resistance mechanisms is increasingly relevant; we surveyed the pan-tumor landscape of *FGFR1-4* genomic alterations [short variants (SVs), gene rearrangements (REs), and copy number alterations (CNAs)], including their association with tumor mutational burden (TMB) and the genomic comutational landscape.

**Patients and methods:**

Comprehensive genomic profiling of 355 813 solid tumor clinical cases was performed using the FoundationOne and FoundationOne CDx assays (Foundation Medicine, Inc.) to identify genomic alterations in >300 cancer-associated genes and TMB (determined on ≤1.1 megabases of sequenced DNA).

**Results:**

*FGFR1-4* SVs and REs occurred in 9603/355 813 (2.7%), and CNAs in 15 078/355 813 (4.2%) samples. Most common *FGFR* alterations for bladder cancer, intrahepatic cholangiocarcinoma, and glioma were *FGFR3* SVs (1051/7739, 13.6%), *FGFR2* REs (618/6641, 9.3%), and *FGFR1* SVs (239/11 550, 2.1%), respectively. We found several, potentially clinically relevant, tumor-specific associations between *FGFR1-4* genomic alterations and other genomic markers. *FGFR3* SV-altered bladder cancers and *FGFR1* SV-altered gliomas were significantly less likely to be TMB-high versus unaltered samples. *FGFR3* SVs in bladder cancer significantly co-occurred with *TERT* and *CDKN2A/B* alterations; *TP53* and *RB1* alterations were mutually exclusive. In intrahepatic cholangiocarcinoma, *FGFR2* REs significantly co-occurred with *BAP1* alterations, whereas *KRAS*, *TP53*, *IDH1*, and *ARID1A* alterations were mutually exclusive. *FGFR1* SVs in gliomas significantly co-occurred with *H3-3A* and *PTPN11* alterations, but were mutually exclusive with *TERT*, *EGFR*, *TP53*, and *CDKN2A/B* alterations.

**Conclusions:**

Overall, our hypothesis-generating findings may help to stratify patients in clinical trials and guide optimal targeted therapy in those with *FGFR* alterations.

## Highlights

• We surveyed the pan-tumor landscape of *FGFR1-4* genomic alterations (SVs, REs, and CNAs).
• In bladder cancers, *FGFR3* SVs were significantly associated with *CDKN2A/B* and *TERT* alterations, and a lower TMB.
• *FGFR2* REs in intrahepatic cholangiocarcinoma co-occurred with *BAP1* alterations but were mutually exclusive with *TP53.*
• *FGFR1* SVs in glioma co-occurred with *H3-3A* alterations.
• Our hypothesis-generating findings may help to guide optimal targeted therapy in those with *FGFR* alterations.

## Introduction

The fibroblast growth factor receptor (FGFR) family consists of four transmembrane receptor proteins, FGFR1-4.^1^ Binding of their respective fibroblast growth factor ligands results in receptor dimerization and activation of downstream signaling pathways [e.g. extracellular-signal regulated kinase (ERK)/mitogen-activated protein kinase (MAPK)], which promotes cell survival, proliferation, development, angiogenesis, and differentiation.^1^ Oncogenic alterations in *FGFR1-4*, including short variants (SVs), gene rearrangements (REs; i.e. movement of DNA gene sequences across the genome, which may lead to gene fusions), and copy number alterations (CNAs), are found in ∼7% of all human cancers, most commonly in urothelial, breast, endometrial, squamous lung, and ovarian cancer, and in cholangiocarcinoma.^1^^,^^2^ The pathogenic potential of *FGFR* alterations is both alteration- and tumor type-specific, impacting the likelihood of patient response to FGFR inhibitors, and therefore underscoring the need for further delineation to inform clinical decision-making.

Based on positive results from clinical trials,^3^, ^4^, ^5^ three selective tyrosine kinase inhibitors targeting particular FGFRs have been granted accelerated approval by the Food and Drug Administration (FDA) for treatment of *FGFR*-altered cancers, including erdafitinib for *FGFR2/3*-altered, previously treated, locally advanced, or metastatic urothelial cancer, as well as pemigatinib and infigratinib for previously treated, locally advanced, or metastatic cholangiocarcinoma with *FGFR2* REs.^6^, ^7^, ^8^ Pemigatinib has also been approved/recommended by the European Medicines Agency (EMA), the National Institute for Health and Care Excellence (NICE), and the Chinese National Medical Products Administration for the same indication, and by the Japanese Ministry of Health, Labour and Welfare for patients with unresectable, *FGFR2* RE-altered, biliary tract cancer that has progressed after at least one prior line of systemic therapy.^9^, ^10^, ^11^, ^12^ Futibatinib, the pan-FGFR1-4 inhibitor, has been granted accelerated FDA approval for the treatment of advanced cholangiocarcinoma with *FGFR2* REs after positive phase II clinical data.^13^^,^^14^ Novel agents are being tested in clinical trials; for example, the highly selective FGFR2 inhibitor RLY-4008 in a clinical trial of patients with intrahepatic cholangiocarcinoma (and other advanced solid tumors) and *FGFR2* alterations (NCT04526106). Initial results suggest potent and selective FGFR2 inhibition and encouraging antitumor activity in FGFR inhibitor-naïve patients, across all doses.^15^ The activity profile of FGFR inhibitors varies according to the type of *FGFR* alteration, with greater objective response rate and progression-free survival associated with SVs and REs, compared with CNAs.^16^^,^^17^ Some patients may also develop resistance to FGFR inhibitors due to mutations arising in the ‘gatekeeper’ residues of FGFR1-4, which are responsible for controlling access of FGFR inhibitors to the receptor.^1^ Resistance can further occur via mutations in other regions of the kinase domain of the receptor: for example, *N550H* in *FGFR2*, which acts as a molecular brake that restricts the kinase to an uncontrolled, active state.^18^

Clinically validated, comprehensive genomic profiling (CGP)-based assays are critical to identify patients who may benefit most from FGFR inhibitors (or other targeted therapies).^1^^,^^19^ CGP is a next-generation sequencing-based method that detects novel and known variants of the four main classes of genomic alterations (insertions and deletions, REs, CNAs, and substitutions), and genomic signatures such as tumor mutational burden (TMB), microsatellite instability, and genome-wide loss of heterozygosity (for patients with ovarian cancer to provide prognostic, diagnostic, and predictive insights that inform research or treatment decisions for individual patients across all cancer types).^20^ The incidence of *FGFR1-4* genomic alterations across different tumor types and the genomic comutational landscape influencing the response to FGFR inhibitors should be studied in a broad manner. This is particularly valuable for tumor types other than cholangiocarcinoma and urothelial carcinoma, in which *FGFR1-4* alterations have been described, but their prevalence and distribution are not well understood. Therefore, we aimed to comprehensively survey the pan-tumor landscape of *FGFR1-4* genomic alterations (SVs, REs, and CNAs), including overall and disease-specific prevalence. For commonly occurring *FGFR1-4* SVs and REs, we also describe their tumor type-specific association with TMB and the genomic comutational landscape, including significantly co-occurring and mutually exclusive (i.e. significantly likely to not co-occur) genomic alterations. This should help to inform molecular-based patient stratification for future clinical trials, next-generation FGFR inhibitor development, and combination therapy for *FGFR*-altered tumors.

## Patients and methods

CGP of 355 813 solid tumor clinical cases (as diagnosed by the treating physician and confirmed on hematoxylin- and eosin-stained slides) was performed using the FoundationOne (F1) and F1CDx assays (Foundation Medicine, Inc., Cambridge, MA, USA), as described previously,^21^^,^^22^ in a Clinical Laboratory Improvement Amendments-certified and College of American Pathologists-accredited laboratory. Regarding urinary tract cancer, we assessed tumors originating from the bladder, urethra, and the upper urinary tract, including urothelial carcinoma and variant histologies. Whereas bladder cancer refers to all cancers originating from the bladder, urinary tract cancers are those originating from areas of the urinary tract other than the bladder, such as the ureter, urethra, and urachus regions.

All samples submitted for sequencing featured a minimum of 20% tumor cell nuclear area and yielded a minimum of 50 ng of extracted DNA. CGP was performed on hybrid-capture, adapter ligation-based libraries, to identify genomic alterations [base substitutions, small insertions and deletions, CNAs (gene copy number of ≥ specimen ploidy +4), and REs] in coding exons (F1CDx: *n* = 309; F1: *n* = 395) and select introns of cancer-associated genes (F1CDx: *n* = 36; F1: *n* = 31), and TMB.^23^ TMB was calculated as the number of nondriver somatic coding mutations per megabase (mut/Mb) of genome sequenced; TMB-high was defined as ≥10 mut/Mb and TMB-low as <10 mut/Mb.

All genomic alterations studied included only those described as functional or pathogenic in the literature and seen in the Catalogue Of Somatic Mutations In Cancer (COSMIC) repository,^24^ or those with a likely functional status (frameshift or truncation events in tumor suppressor genes). Variants of unknown significance were not studied.

As self-reported race was not available, genomic ancestry was determined for each patient sample. For each profiling platform (F1 and F1CDx), >40 000 single-nucleotide polymorphism sites sequenced by CGP were identified. To remove biases due to linkage disequilibrium, linkage pruning was performed using PLINK (using the –indep flag with a window size of 50, a step size of 5, and a variance inflation factor threshold of 2). A random forest classifier was trained on the 1000 Genomes Project samples to identify ancestral populations [African, Admixed American (Hispanic), East Asian, South Asian] using genetic variation at the single-nucleotide polymorphism sites. Genetic variation was defined by 10 features that captured allele-count variation as determined by principal component analysis. This classifier was applied to CGP patient samples to assign them to one of the ancestral populations.

All statistical analyses were performed using R software v4.0.3 (R Foundation for Statistical Computing, Vienna, Austria) and Python v.2.7.16 (Python Software Foundation, Wilmington, DE, USA). Proportions of categorical variables were compared using Fisher’s exact test. Wilcoxon rank sum was used to test for differences between continuous variables. All *P* values were two-sided, and multiple hypothesis testing correction was performed using the Benjamini–Hochberg procedure to calculate the false discovery rate.

Approval for this analysis, including a waiver of informed consent and a Health Insurance Portability and Accountability Act waiver of authorization, was obtained from the Western Institutional Review Board (Protocol No. 20152817).

## Results

### Overall prevalence of *FGFR1-4* genomic alterations and baseline demographics

We first assessed the broad landscape of *FGFR1-4* SVs and REs across all tumor types. CNAs were considered separately due to limited clinical evidence for their utility as predictive biomarkers of response to FGFR inhibitors.^16^^,^^17^ Of the 355 813 solid tumor samples, 195 155 (54.8%) were from females and 273 710 (76.9%) were of European ancestry. *FGFR1-4* SVs and REs were observed in 9603/355 813 (2.7%) samples (Table 1). *FGFR1*, *FGFR2*, *FGFR3*, and *FGFR4* SVs and REs were seen in 998 (0.3%), 4553 (1.3%), 3800 (1.1%), and 303 (0.09%) samples, respectively (Table 1). The pan-cancer prevalence of partner genes for *FGFR1-4* REs is shown in Supplementary Table S1, available at https://doi.org/10.1016/j.esmoop.2022.100641; the most common partner gene for *FGFR1-4* REs was *TACC1* (71/279, 25.5%), *BICC1* (288/1854, 15.5%), *TACC3* (954/1158, 82.4%), and *NSD1* (5/11, 45.5%), respectively.

**Table 1:** Pan-cancer prevalence of *FGFR1-4* SVs, REs, and CNAs

Gene	Prevalence (*N* = 355 813), *n* (%)
*FGFR1*	
Overall	12 784 (3.6)
RE	279 (0.08)
SV	719 (0.2)
CNA	11 869 (3.3)
*FGFR2*	
Overall	6025 (1.7)
RE	1854 (0.5)
SV	2735 (0.8)
CNA	1707 (0.5)
*FGFR3*	
Overall	4748 (1.3)
RE	1158 (0.3)
SV	2683 (0.8)
CNA	1152 (0.3)
*FGFR4*	
Overall	871 (0.2)
RE	11 (0.003)
SV	292 (0.08)
CNA	570 (0.2)

CNA, copy number alteration; *FGFR*, fibroblast growth factor receptor; RE, gene rearrangement; SV, short variant.

Overall, samples with *FGFR1-4* SVs and REs, versus the unaltered samples, were significantly less likely to be of metastatic origin (2961/7666, 38.6%, versus 127 292/282 631, 45.0%; *P* = 4.2 × 10^−29^), and significantly more likely to be from older patients [median age: 65.0 years, interquartile range (IQR) 56.0-73.0 versus 64.0 years, IQR 55.0-72.0; *P* = 2.6 × 10^−16^] and those of European ancestry (7563/9603, 78.7%, versus 266 147/346 210, 76.8%; *P* = 1.4 × 10^−5^; Supplementary Table S2, available at https://doi.org/10.1016/j.esmoop.2022.100641).

*FGFR1-4* CNAs were observed in 15 078/355 813 (4.2%) samples (Table 1); *FGFR1*, *FGFR2*, *FGFR3*, and *FGFR4* CNAs were observed in 11 869 (3.3%), 1707 (0.5%), 1152 (0.3%), and 570 (0.2%) samples, respectively (Table 1).

### Disease-specific prevalence of *FGFR1-4* genomic alterations

**Figure 1:**
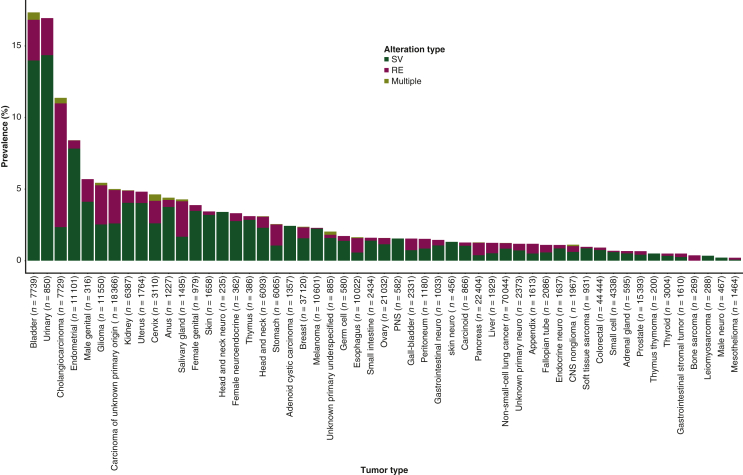
**Disease-specific prevalence of *FGFR1-4* SVs and REs. Specimens with at least one SV and RE in any one of *FGFR1-4* are included in the ‘Multiple’ group.** Only tumor types with an overall incidence of ≥100 patients are shown. CNS, central nervous system; *FGFR*, fibroblast growth factor receptor; PNS, peripheral nervous system; RE, gene rearrangement; SV, short variant.

Across disease types, *FGFR1-4* SVs and REs were most commonly observed in bladder cancer (SVs: 1082/7739, 14.0%; REs: 220/7739, 2.8%), urinary tract cancer (SVs: 122/850, 14.4%; REs: 22/850, 2.6%), cholangiocarcinoma (SVs: 181/7729, 2.3%; REs: 668/7729, 8.6%), and endometrial cancers (SVs: 869/11 101, 7.8%; REs: 63/11 101, 0.6%); in bladder cancer, urinary tract cancer, and endometrial cancer, these were mostly SVs, whereas in cholangiocarcinoma, REs were the predominant *FGFR* genomic alteration (Figure 1).

CNAs most commonly occurred in breast (4907/37 120, 13.2%), uterine (96/1764, 5.4%), and female neuroendocrine cancer (encompasses neuroendocrine carcinomas of the breast, cervix, ovary, uterus, vagina, and vulva; 22/362, 6.1%), with most CNAs being detected in *FGFR1* (Supplementary Figure S1, available at https://doi.org/10.1016/j.esmoop.2022.100641).

**Figure 2:**
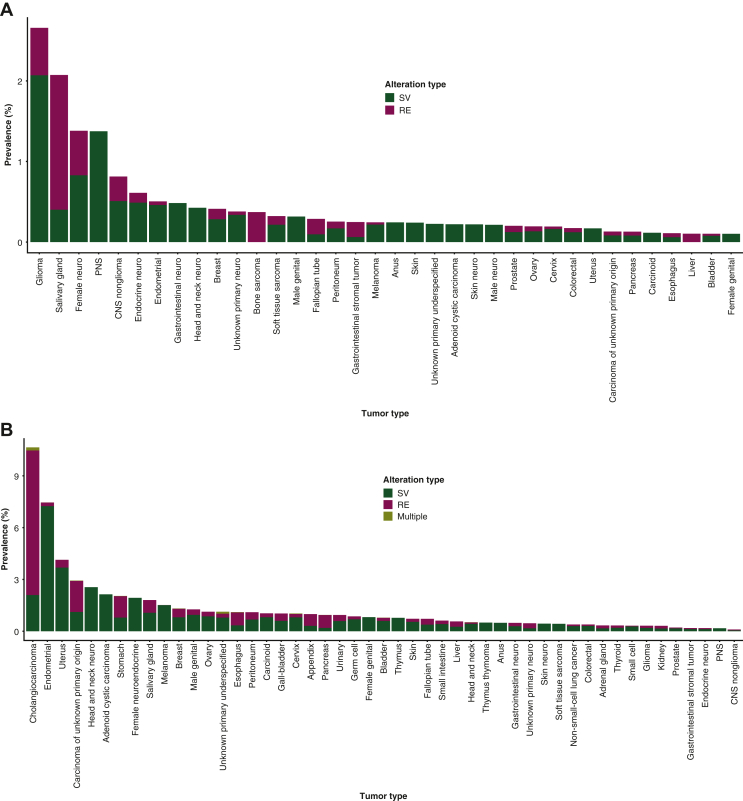

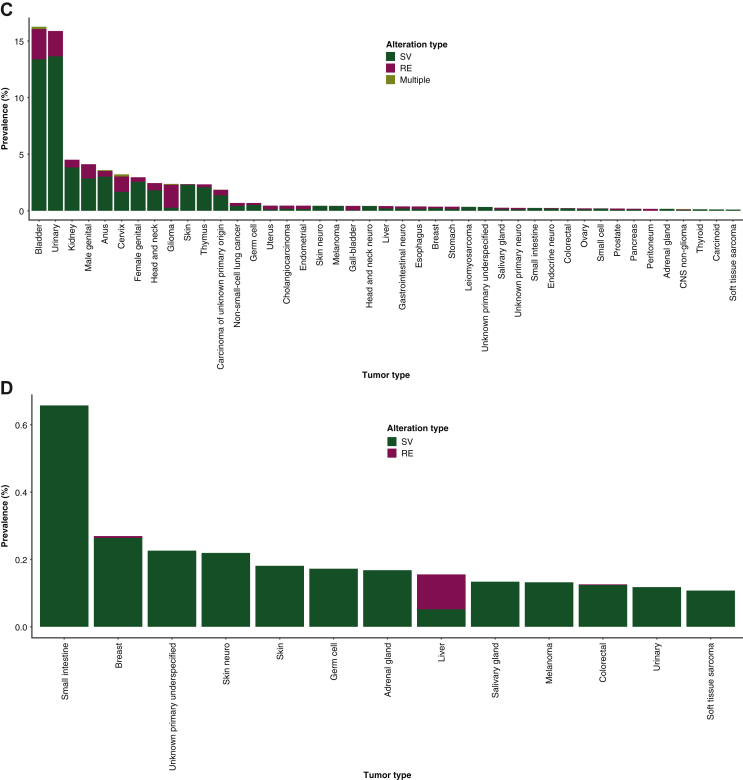
**Disease-specific prevalence of SVs and REs in (A) *FGFR1*, (B) *FGFR2*, (C) *FGFR3*, and (D) *FGFR4*.** Specimens with at least one SV and RE in any one of *FGFR1-4* are included in the ‘Multiple’ group. Only tumor types with an overall incidence of ≥100 patients and a total prevalence of *FGFR1/2/3/4* SVs and REs of ≥0.1% are shown. CNS, central nervous system; *FGFR*, fibroblast growth factor receptor; PNS, peripheral nervous system; RE, gene rearrangement; SV, short variant.

*FGFR1* SVs/REs demonstrated the greatest frequency in glioma (SVs: 239/11 150, 2.1%; REs: 68/11 150, 0.6%), salivary gland cancer (SVs: 6/1495, 0.4%; REs: 25/1495, 1.7%), and female-neuroendocrine cancers (SVs: 3/362, 0.8%; REs: 2/362, 0.6%; Figure 2A). By contrast, *FGFR2* SVs/REs most commonly occurred in cholangiocarcinoma (SVs: 162/7729, 2.1%; REs: 647/7729, 8.4%), specifically intrahepatic cholangiocarcinoma (SVs: 159/6641, 2.4%; REs: 618/6641, 9.3%), extrahepatic cholangiocarcinoma (SVs: 16/993, 1.6%; REs: 39/993, 3.9%), and mixed hepatocellular carcinoma (SVs: 1/95, 1.1%; REs: 4/95, 4.2%; Figure 2B). *FGFR2* SVs/REs also commonly occurred in endometrial cancer (SVs: 804/11 101, 7.2%; REs: 23/11 101, 0.2%; Figure 2B).

*FGFR3* SVs/REs were most frequent in bladder cancer (SVs: 1051/7739, 13.6%; REs: 207/7739, 2.7%) and urinary tract cancer (SVs: 116/850, 13.7%; REs: 19/850, 2.2%; Figure 2C). Specifically, among urinary tract cancers, *FGFR3* SVs/REs were seen in tumors originating from the upper urinary tract (285/850, 33.5%; SVs: 50/285, 17.5%; REs: 6/285, 2.1%). *FGFR4* SVs/REs most commonly occurred in small intestinal cancer (SVs: 16/2434, 0.7%; REs: 0; Figure 2D). In the majority of cases, genomic alterations mainly comprised SVs, except for *FGFR1* alterations in salivary gland carcinoma and *FGFR2* alterations in cholangiocarcinoma, in which REs were most common (Figure 2). Thus, the pattern of SVs and REs detected varied depending on the *FGFR* gene and disease being studied.

### Pan-cancer prevalence of *FGFR1-4* SVs

Lollipop plots demonstrating the positional distribution of the amino acid changes of *FGFR1-4* SVs across all cancer types are shown in Supplementary Figure S2, available at https://doi.org/10.1016/j.esmoop.2022.100641. For each protein, the top five codon-level amino acid changes are annotated. These include *FGFR1*: N546K (protein kinase domain; 334/355 813, 0.09%), K656E (protein kinase domain; 126/355 813, 0.04%), D133N/_S134insD/del (36/355 813, 0.01%), R445W (42/355 813, 0.01%), and V561L/M (protein kinase domain; 33/355 813, 0.009%); *FGFR2*: S252A/C/P/T/W (680/355 813, 0.2%), N549D/H/I/K/S/T/Y/_L550>KI (protein kinase domain; 460/355 813, 0.13%), C382F/R/_I383>R (286/355 813, 0.08%), K659E/M/N (protein kinase domain; 219/355 813, 0.06%), and Y375C (149/355 813, 0.04%); *FGFR3*: S249C/F/Y (1350/355 813, 0.38%), R248C/S/_S249del/_S249insC (351/355 813, 0.1%), Y373C/H/N (308/355 813, 0.09%), G380E/R (119/355 813, 0.03%), and K650E/M/N/Q/T (protein kinase domain; 107/355 813, 0.03%); *FGFR4*: V510A/E/G/L (protein kinase domain; 37/355 813, 0.01%), R80W (22/355 813, 0.006%), R394Q (21/355 813, 0.006%), R610H (protein kinase domain; 15/355 813, 0.004%), and D135N (12/355 813, 0.003%), demonstrating the diversity of activating *FGFR* mutations not only in the protein kinase domain, but also across the transmembrane and extracellular domains.

### Comutational landscape and mutual exclusivity of *FGFR1-4* genomic alterations

We studied the baseline demographics of commonly occurring *FGFR* SVs and REs in bladder cancer, cholangiocarcinoma, and glioma. These were selected based on the prevalence of *FGFR* SVs and REs and emerging clinical interest (e.g. for gliomas). We also assessed the disease-specific alteration type, comutational landscape, and mutual exclusivity of these cancer types, as well as urinary tract cancer.

**Figure 3:**
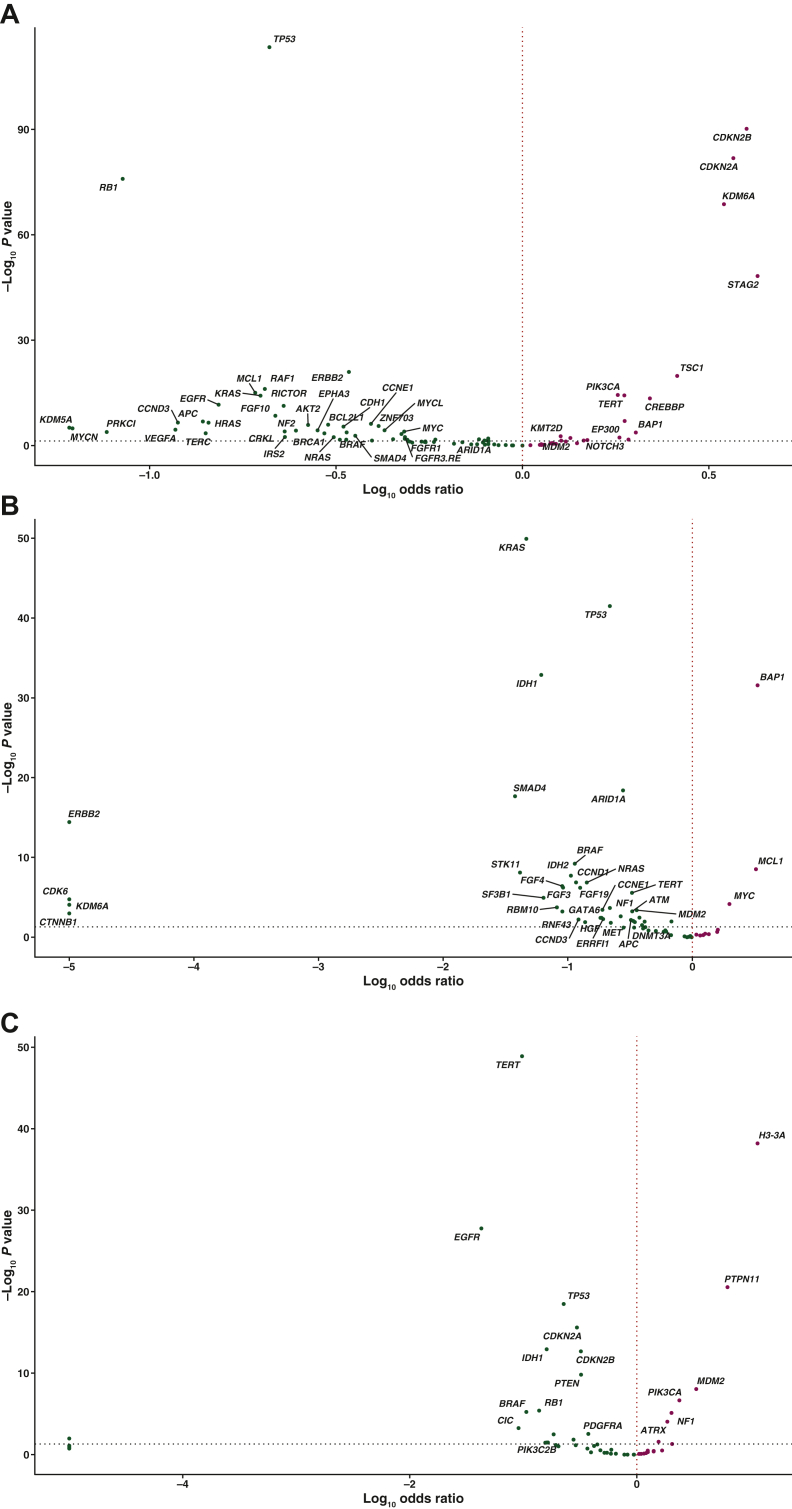
**Significant co-occurrence and mutual exclusivity of (A) *FGFR3* SVs in bladder cancer, (B) *FGFR2* REs in intrahepatic cholangiocarcinoma, and (C) *FGFR1* SVs in glioma.** Red and Green dots indicate genomic alterations co-occurring and mutually exclusive with the *FGFR* alteration, respectively. Two-tailed Fisher’s exact test was used to estimate the *P* values and odds ratio of associations between genomic alterations and the *FGFR* alteration. The Benjamini–Hochberg procedure was used to estimate the adjusted *P* values. Only genes with a disease prevalence ≥1% were included, and only genes with an adjusted *P* value ≤0.05 were labeled. *FGFR*, fibroblast growth factor receptor; PIK3CA, phosphatidylinositol-4,5-bisphosphate 3-kinase, catalytic subunit alpha; RE, gene rearrangement; SV, short variant.

In bladder cancer, genomic alterations were most common in *FGFR3*, particularly in the form of SVs. *FGFR3* SV-altered bladder cancers were significantly more likely to be from older patients (*P* = 4.5 × 10^−5^; Supplementary Table S3, available at https://doi.org/10.1016/j.esmoop.2022.100641) and less likely to be TMB-high [260/1051, 24.7%, versus 2336/6688, 34.9%; odds ratio = 1.63; *P* = 3.49 × 10^−11^; median TMB 6.09 (IQR 3.48-9.57) versus 6.25 (IQR 3.75-12.50); Supplementary Figure S3, available at https://doi.org/10.1016/j.esmoop.2022.100641], versus unaltered samples. We observed significant co-occurrence of *FGFR3* SVs with *TERT* (*TERT* prevalence in *FGFR3* SV-altered: 858/1051, 81.6%, versus *TERT* prevalence in *FGFR3* SV-unaltered: 4703/6688, 70.3%; odds ratio = 1.88; *P* = 5.22 × 10^−15^) and *CDKN2A/B* alterations (671/1051, 63.8%, versus 2189/6688, 32.7%; odds ratio = 3.63; *P* = 9.0 × 10^−81^; Figure 3A); alterations in *TP53* (310/1051, 29.5%, versus 4456/6688, 66.6%; odds ratio = 0.21; *P* = 3.94 × 10^−114^) and *RB1* (27/1051, 2.6%, versus 1588/6688, 23.7%; odds ratio = 0.08; *P* = 1.19 × 10^−76^) were found to be significantly mutually exclusive with *FGFR3* SVs (Figure 3A). *FGFR3* SVs were also common in urinary tract cancer and *FGFR3* SV-altered urinary tract cancers were significantly more likely to be TMB-high versus unaltered samples [34/116, 29.3%, versus 131/734, 17.9%; odds ratio = 0.52; *P* = 0.005; median TMB 5.22 (IQR 2.5-10.44) versus 5.00 (IQR 2.5-7.5); Supplementary Figure S4A, available at https://doi.org/10.1016/j.esmoop.2022.100641]. Co-occurrence and mutual exclusivity findings were similar to those found for *FGFR3* SV-altered bladder cancers (Supplementary Figure S4B, available at https://doi.org/10.1016/j.esmoop.2022.100641).

In cholangiocarcinoma, of which intrahepatic was the most common type, *FGFR2* alterations were the most frequent genomic alteration, with REs comprising the majority. *FGFR2* RE-altered intrahepatic cholangiocarcinomas were significantly more likely to be from younger, female patients of African or American ancestry with tumors of local origin (*P* = 4.6 × 10^−10^, <2.3 × 10^−16^, 0.001, 0.02, and 1.7 × 10^−12^, respectively; Supplementary Table S3, available at https://doi.org/10.1016/j.esmoop.2022.100641) and less likely to be TMB-high [3/618, 0.5%, versus 243/6022, 4.0%; odds ratio = 8.62; *P* = 1.34 × 10^−7^; median TMB 1.74 (IQR 0.87-2.5) versus 1.74 (IQR 0.37-3.75); Supplementary Figure S5, available at https://doi.org/10.1016/j.esmoop.2022.100641], versus their *FGFR2* RE-unaltered counterparts. *BAP1* alterations (*BAP1* prevalence in *FGFR2* RE-altered: 192/618, 31.1%, versus *BAP1* prevalence in *FGFR2* RE-unaltered: 717/6023, 11.9%; odds ratio = 3.34; *P* = 2.73 × 10^−32^) significantly co-occurred with *FGFR2* REs, whereas *KRAS* (8/618, 1.3%, versus 1325/6023, 22.0%; odds ratio = 0.05; *P* = 1.21 × 10^−50^), *TP53* (67/618, 10.8%, versus 2159/6023, 35.8%; odds ratio = 0.22; *P* = 3.24 × 10^−42^), *IDH1* (7/618, 1.1%, versus 949/6023, 15.8%; odds ratio = 0.06; *P* = 1.36 × 10^−33^), and *ARID1A* alterations (39/618, 6.3%, versus 1176/6023, 19.5%; odds ratio = 0.28; *P* = 4.02 × 10^−19^) were found to be significantly mutually exclusive (Figure 3B).

*FGFR1-4* alterations in glioma were mainly *FGFR1* SVs. *FGFR1* SV-altered gliomas were significantly more likely to be from younger patients of American or European ancestry (*P* <2.2 × 10^−16^, = 0.007, and = 0.002, respectively; Supplementary Table S3, available at https://doi.org/10.1016/j.esmoop.2022.100641) and less likely to be from tumors of a local origin (*P* = 3.3 × 10^−8^), versus unaltered gliomas. No significant difference existed in TMB between *FGFR1* SV-altered versus SV-unaltered gliomas [TMB-high: 5/239, 2.1%, versus 460/11 311, 4.1%; odds ratio = 1.98; *P* = 0.14; median TMB 1.25 (IQR 0.87-2.61) versus 1.74 (IQR 0.87-3.48); Supplementary Figure S6, available at https://doi.org/10.1016/j.esmoop.2022.100641]. *H3-3A* (*H3-3A* prevalence in *FGFR1* SV-altered: 64/239, 26.8%, versus *H3-3A* prevalence in *FGFR1* SV-unaltered: 346/11 311, 3.1%; odds ratio = 11.59; *P* = 6.37 × 10^−39^) and *PTPN11* alterations (51/239, 21.3%, versus 467/11 311, 4.1%; odds ratio = 6.30; *P* = 2.81 × 10^−21^) were found to be significantly co-occurring with *FGFR1* SVs, whereas alterations in *TERT* (29/239, 12.1%, versus 6629/11 311, 58.6%; odds ratio = 0.10; *P* = 1.27 × 10^−49^), *EGFR* (4/239, 1.7%, versus 3222/11 311, 28.5%; odds ratio = 0.04; *P* = 1.74 × 10^−28^), *TP53* (31/239, 13.0%, versus 4482/11 311, 39.6%; odds ratio = 0.23; *P* = 3.26 × 10^−19^), and *CDKN2A/B* (48/239, 20.1%, versus 5197/11 311, 45.9%; odds ratio = 0.30; *P* = 1.6 × 10^−16^) were observed to be significantly mutually exclusive (Figure 3C).

**Figure 4:**
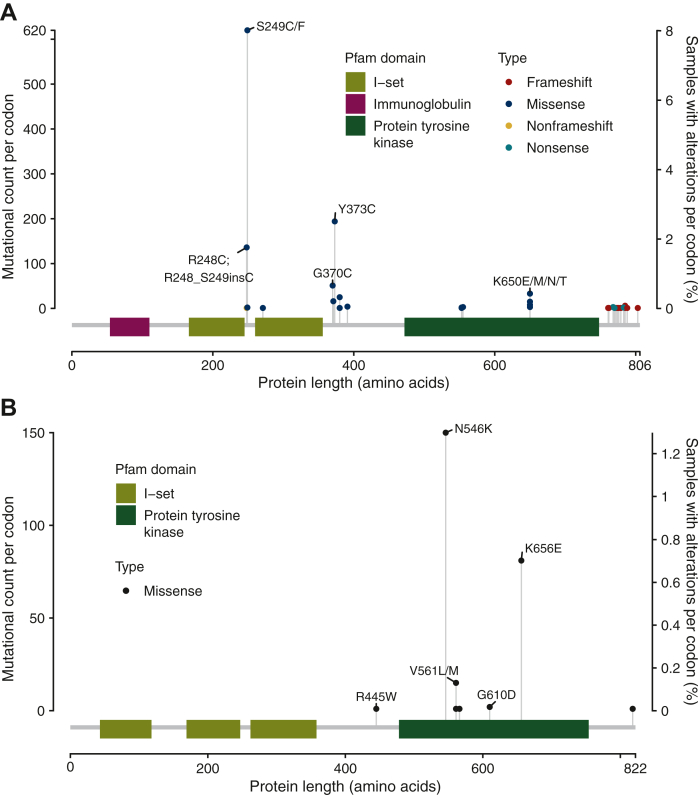
**Positional distribution of the amino acid changes, with the top five most frequently altered codons and a schematic of the protein architecture annotated, of (A) *FGFR3* SVs in bladder cancer and (B) *FGFR1* SVs in glioma.** Multiple single amino acid substitutions at the same codon are ordered alphabetically, not by frequency. Complex alterations, such as indels, are listed after substitutions if present at the same codon for clarity. *FGFR*, fibroblast growth factor receptor; SV, short variant.

We also analyzed the positional distribution of the amino acid changes of *FGFR3* SVs in bladder cancer and *FGFR1* SVs in glioma. In bladder cancer, the top five most frequently observed *FGFR3* altered codons with SVs and their amino acid changes were S249C/F (620/7739, 8.0%), Y373C (194/7739, 2.5%), R248C_S249insC (136/7739, 1.8%), G370C (51/7739, 0.7%), and K650E/M/N/T (33/7739, 0.4%; Figure 4A), with the most commonly altered hotspot S249 being observed in the extracellular region of *FGFR3*. For *FGFR1* SVs in glioma, this was N546K (150/11 550, 1.3%), K656E (81/11 550, 0.7%), V561L/M (15/11 550, 0.1%), G610D (2/11 550, 0.02%), and R445W (1/11 550, 0.009%; Figure 4B), with the most commonly altered hotspot N546 being observed in the protein kinase domain.

## Discussion

To our knowledge, this study is the largest to date that comprehensively describes the pan-tumor landscape of *FGFR1-4* genomic alterations (SVs, REs, and CNAs), including overall and disease-specific prevalence and disease-specific genomic comutational landscape. In this study, *FGFR1-4* SVs/REs were seen in 2.7% of samples (mostly SVs; *FGFR4* alterations made up only a small proportion). As reported previously,^2^^,^^25^^,^^26^ CNAs were detected in a higher proportion (4.2%) and were most common in *FGFR1*, and SVs/REs were most common in *FGFR2/3*.

Overall, in our study, samples with *FGFR1-4* SVs and REs, versus the unaltered samples, were more likely to be of European ancestry. This may represent a general trend, especially given that *FGFR2* REs are more common in patients of European versus Asian descent,^19^ although further study with detailed clinical data is required to elucidate whether this impacts patient outcomes. Furthermore, there are more significant socioeconomic variables involved, so it is difficult to draw conclusions.

Consistent with previous studies,^2^^,^^26^ we found that CNAs occurred most commonly in breast cancer. The usefulness of CNAs as a patient selection tool for FGFR inhibitors is still not well understood, particularly considering the low concordance rate between *FGFR1* amplification, the most common type of *FGFR* CNA, and *FGFR1* messenger RNA (and thus protein) expression.^27^ However, a translational clinical trial demonstrated high-level *FGFR2* amplification to predict response to the FGFR inhibitor AZD4547 in gastric cancer^28^; thus the association of CNAs with response to FGFR inhibitors may be gene- and tumor type-specific. In this trial, only high-level CNAs were studied; clinical analyses of the response to inhibitors in patients with high-level *FGFR* CNAs are therefore required.^28^

*FGFR3* alterations were most common in bladder and urinary tract cancer in this study, with the majority being SVs. A high prevalence of *FGFR3* SVs in urothelial cancers, with an enrichment in tumors originating from the renal pelvis and ureter, has been detected previously.^2^ The anti-FGFR therapy erdafitinib has shown clinical effectiveness in these tumor types and has subsequently been approved by the FDA.^3^^,^^6^^,^^29^ However, the response rates reported with other pan-FGFR inhibitors (≤25%) were substantially lower compared with that of erdafitinib (40%).^29^, ^30^, ^31^, ^32^, ^33^ These differences may be partly related to the differing properties of the inhibitors (e.g. erdafitinib has a relatively long half-life) and molecular characteristics of the tumors from the varying patient populations. Indeed, we also found in these cancers a significant co-occurrence of *FGFR3* SVs with *CDKN2A*/*B* and *TERT*, as has been found previously,^33^ suggesting that it may be possible to stratify patients according to these alterations, but clinical data are required to confirm this hypothesis. *Phosphatidylinositol-4,5-bisphosphate 3-kinase, catalytic subunit alpha (PIK3CA)* alterations have also been detected in *FGFR3*-altered urothelial cancers^2^; however, combined phosphoinositide 3-kinase and FGFR inhibition with alpelisib and infigratinib, respectively, in *PIK3CA*-altered solid tumors has not shown clear evidence of synergistic activity, and potential toxicity is a concern.^34^

In our study, *FGFR3* SVs in bladder cancer were significantly associated with a lower TMB. This is consistent with the fact that *FGFR3* SVs are typically enriched in luminal-type tumors such as urothelial cancer, which are characterized by a depleted T-cell environment and a reduced predicted response to immune checkpoint inhibitors.^35^^,^^36^ Nonetheless, *FGFR3* SVs do not appear to confer resistance to immune checkpoint inhibitors, with multiple studies demonstrating similar outcomes between patients with *FGFR3*-altered and *FGFR3*-unaltered tumors.^36^, ^37^, ^38^ This suggests that combined FGFR and immune checkpoint inhibition may be clinically useful in *FGFR*-altered, metastatic urothelial cancer. Indeed, preclinical analysis has shown FGFR inhibitors to broaden the T-cell repertoire in cancer models through increased CD4+ and CD8+ T-cell infiltration; the same analysis also demonstrated immune checkpoint inhibitors to focus this pre-existing T-cell response through clonal expansion.^39^ Clinically, early data from the NORSE (NCT03473743) and FORT-2 (NCT03473756) trials of pan-FGFR inhibitors combined with immune checkpoint inhibitors in patients with first-line metastatic, *FGFR*-altered urothelial cancer have shown signals of potential synergism with manageable safety profiles.^40^^,^^41^ As TMB has been demonstrated to be a marker of benefit to immune checkpoint inhibitor therapies,^42^^,^^43^ it is possible that future strategies of combination therapy would demonstrate enriched responses in selected populations of patients with TMB-high tumors. Moreover, clinical studies of patients with metastatic urothelial cancer treated with the pan-FGFR inhibitor erdafitinib have shown higher response rates in those previously treated with cancer immunotherapy,^3^^,^^44^ suggesting that FGFR inhibitors may be useful in patients with urothelial cancers who do not respond to cancer immunotherapy.

Our co-occurring mutational analysis of intrahepatic cholangiocarcinoma revealed *FGFR2* REs to associate significantly with *BAP1* alterations, as seen previously.^19^^,^^45^ *BAP1* is a tumor suppressor gene that has epigenetic functions in cancer^46^; however, co-occurring alterations in *BAP1* are not prognostic for overall survival, objective response rate, or progression-free survival in patients with cholangiocarcinoma treated with pemigatinib, the selective FGFR1/2/3 inhibitor.^19^^,^^45^ We also observed *TP53* alterations to be significantly mutually exclusive with *FGFR2* REs. In contrast to *BAP1*, the presence of co-occurring *TP53* alterations in tumors has previously been associated with a shorter overall survival and median progression-free survival in patients with cholangiocarcinoma treated with pemigatinib, compared with those without.^19^^,^^45^ This suggests that patients with alterations in tumor suppressor genes such as *TP53* may have worse outcomes with FGFR inhibitors, warranting further research into combination therapy in these patients. This may also apply to bladder cancers and gliomas, in which *TP53* alterations were also found in this study to be mutually exclusive with *FGFR3* SVs and *FGFR1* SVs, respectively.

We found that the most common *FGFR* alterations in gliomas were *FGFR1* SVs, as seen in other studies (along with *FGFR3* alterations).^2^^,^^25^ A recent clinical trial of infigratinib in patients with recurrent/progressive glioma and any *FGFR* alteration revealed durable disease control lasting >1 year in patients with *FGFR1/3* point mutations or *FGFR3*-*TACC3* fusions.^47^ This indicates that refined future trials of infigratinib, either alone or in combination with other targeted therapies, may be of interest in patients with gliomas harboring specific *FGFR* aberrations. In this study, *FGFR1* SVs in glioma were found to be co-occurring with *H3-3A* and mutually exclusive with *CDKN2A/2B*. The genomic co-occurrence findings here and in other studies therefore suggest that possible combination partners for infigratinib may be those with clinical activity against *H3*-altered gliomas (e.g. ONC2021).^47^^,^^48^ It is also interesting that both intrahepatic cholangiocarcinoma and glioma *FGFR* alterations were significantly associated with alterations in epigenetic modifiers, suggesting a potential mechanistic role in these tumors.

This is the largest study thus far to investigate pan-cancer *FGFR* alterations, with unprecedented coverage of analyzed cases and diverse cohort characteristics, enabling cross-comparison of genomic findings of different tumor types using a single, clinically validated assay. A limitation of the study is the lack of treatment outcome data, preventing analyses of individual patient outcomes and response following therapy. A second limitation is the lack of correlation analyses of circulating tumor DNA with tissue biomarker findings, particularly given that next-generation sequencing-based circulating tumor DNA analysis may be able to reveal additional *FGFR* alterations and therapeutic targets not shown by some forms of tissue-based testing.^49^^,^^50^ Other limitations include a lack of a central pathology review of samples, large time frame for sample collection, potential bias toward European ancestry, and lack of association with other important analyses; for example, *FGFR3* messenger RNA expression or gene expression findings and molecular subtypes.

### Conclusions

In conclusion, we comprehensively surveyed the pan-tumor landscape of *FGFR1-4* genomic alterations in this study and identified a number of co-occurring and mutually exclusive alterations in other genes, as well as associations with the genomic signature TMB. Such hypothesis-generating findings may help to stratify patients in clinical trials and guide optimal targeted therapy in those with *FGFR* alterations.
