# Supplementary material for: Pan-tumor landscape of fibroblast growth factor receptor 1-4 genomic alterations
Source: ESMO Open. 2022 Nov 30;7(6):100641. doi: 10.1016/j.esmoop.2022.100641 (PMC9832751; doi:10.1016/j.esmoop.2022.100641)
Supplement: Supplementary Figures [file mmc1.docx]

**SUPPLEMENTARY FIGURES**

**Supplementary Figure S1. Disease-specific prevalence of *FGFR1-4* CNAs.** Only tumor types with an overall incidence of ≥100 patients are shown. CNA, copy number amplification; *FGFR*, fibroblast growth factor receptor.


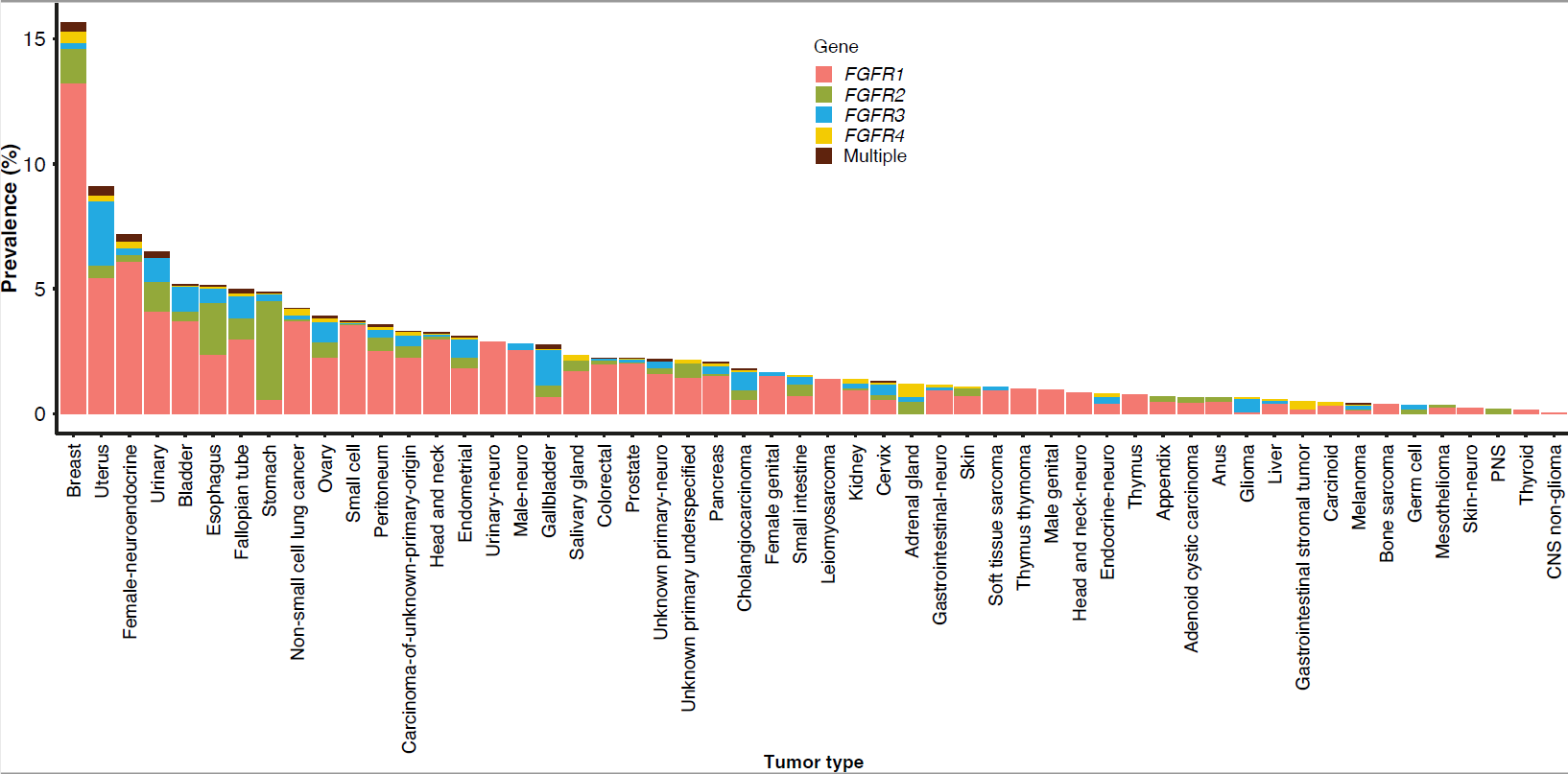


**Supplementary Figure S2. Positional distribution of the amino acid changes of SVs, with the top five most frequently altered codons and a schematic of the protein architecture annotated, in: (A) *FGFR1*, (B) *FGFR2*, (C) *FGFR3*, and (D) *FGFR4*.** Multiple single amino acid substitutions at the same codon are ordered alphabetically, not by frequency. Complex alterations, like indels, are listed after substitutions if present at the same codon for clarity. *FGFR*, fibroblast growth factor receptor; SV, short variant.

**A**


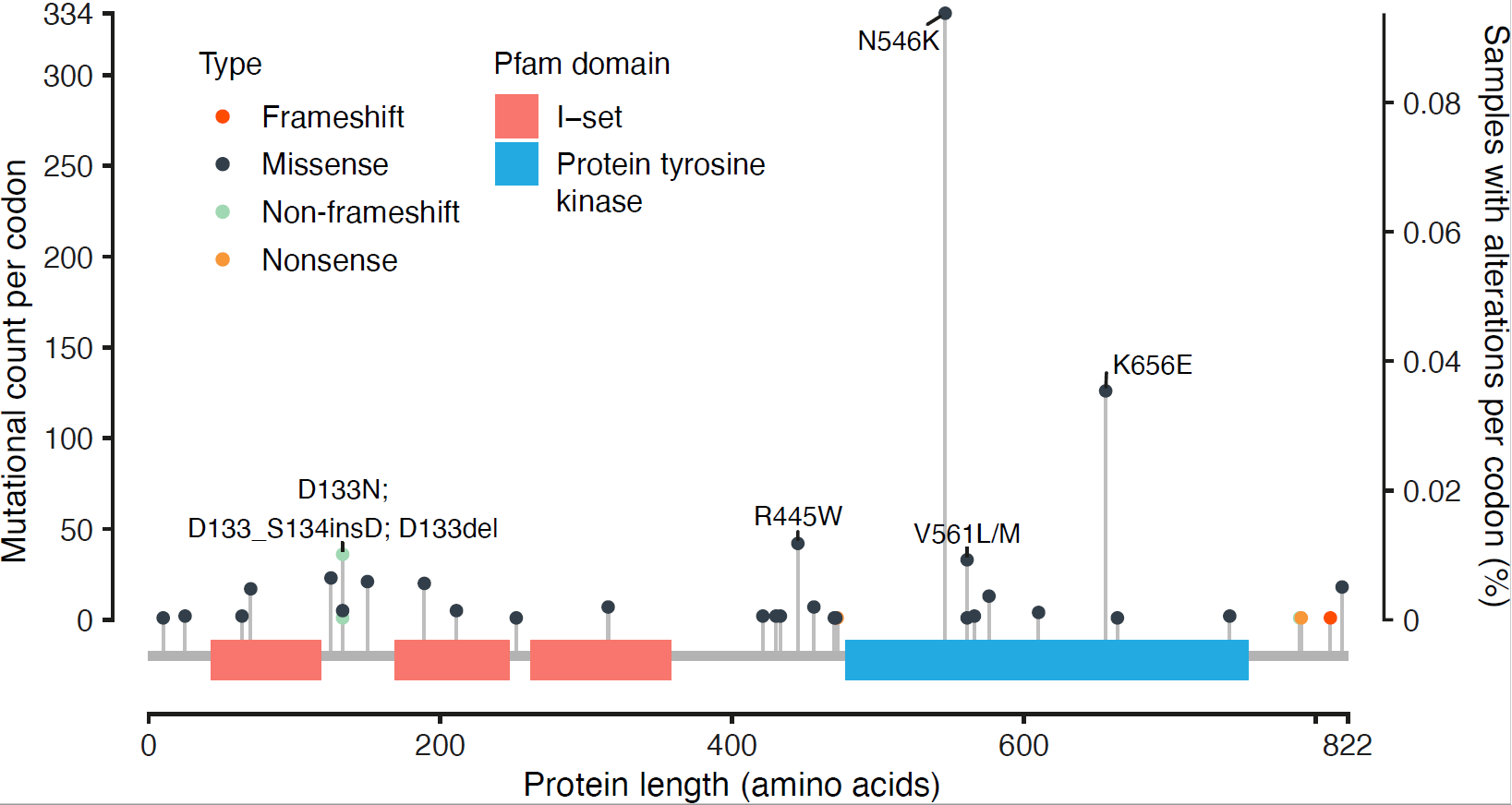


**B**


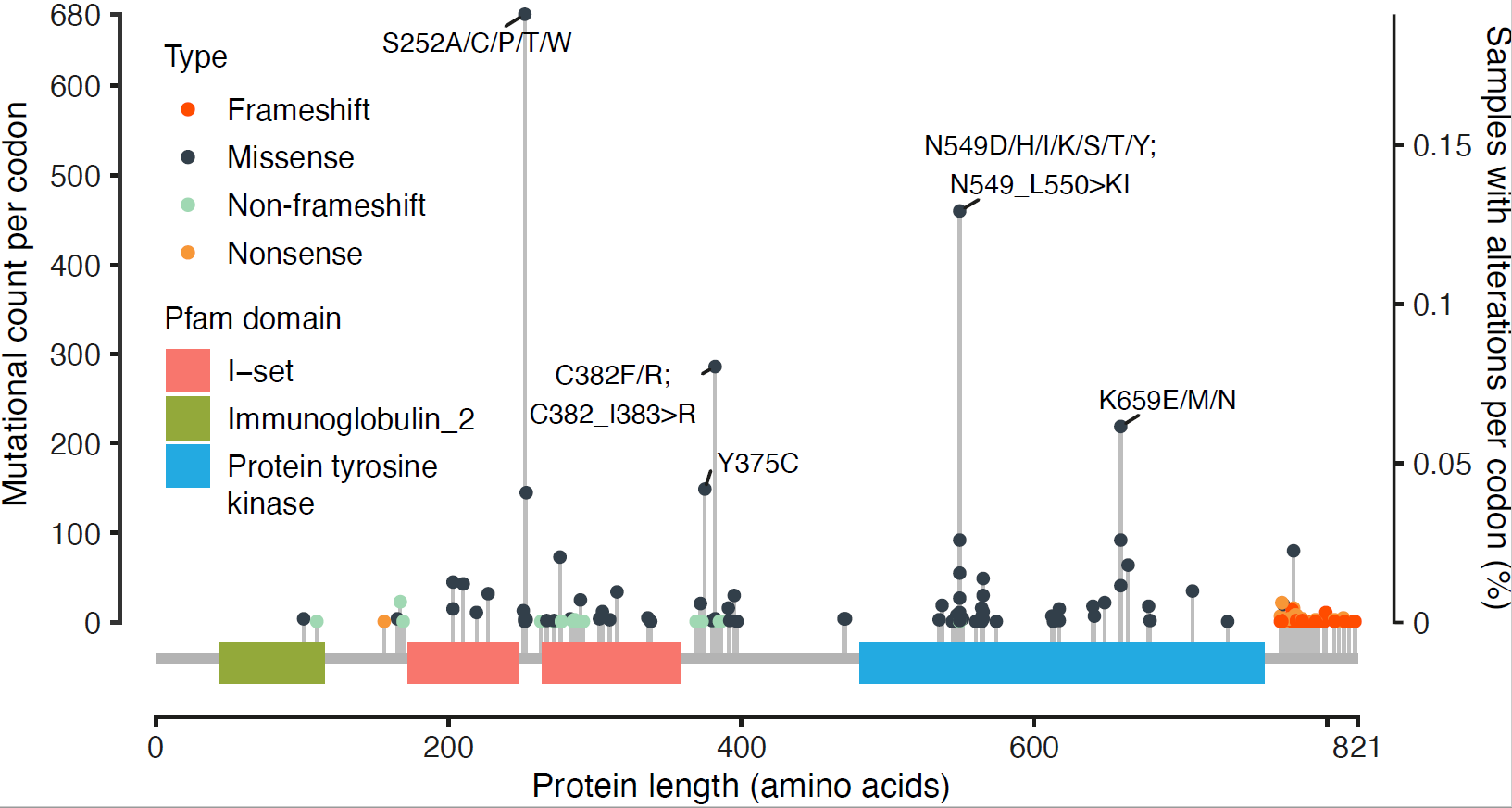


**C**


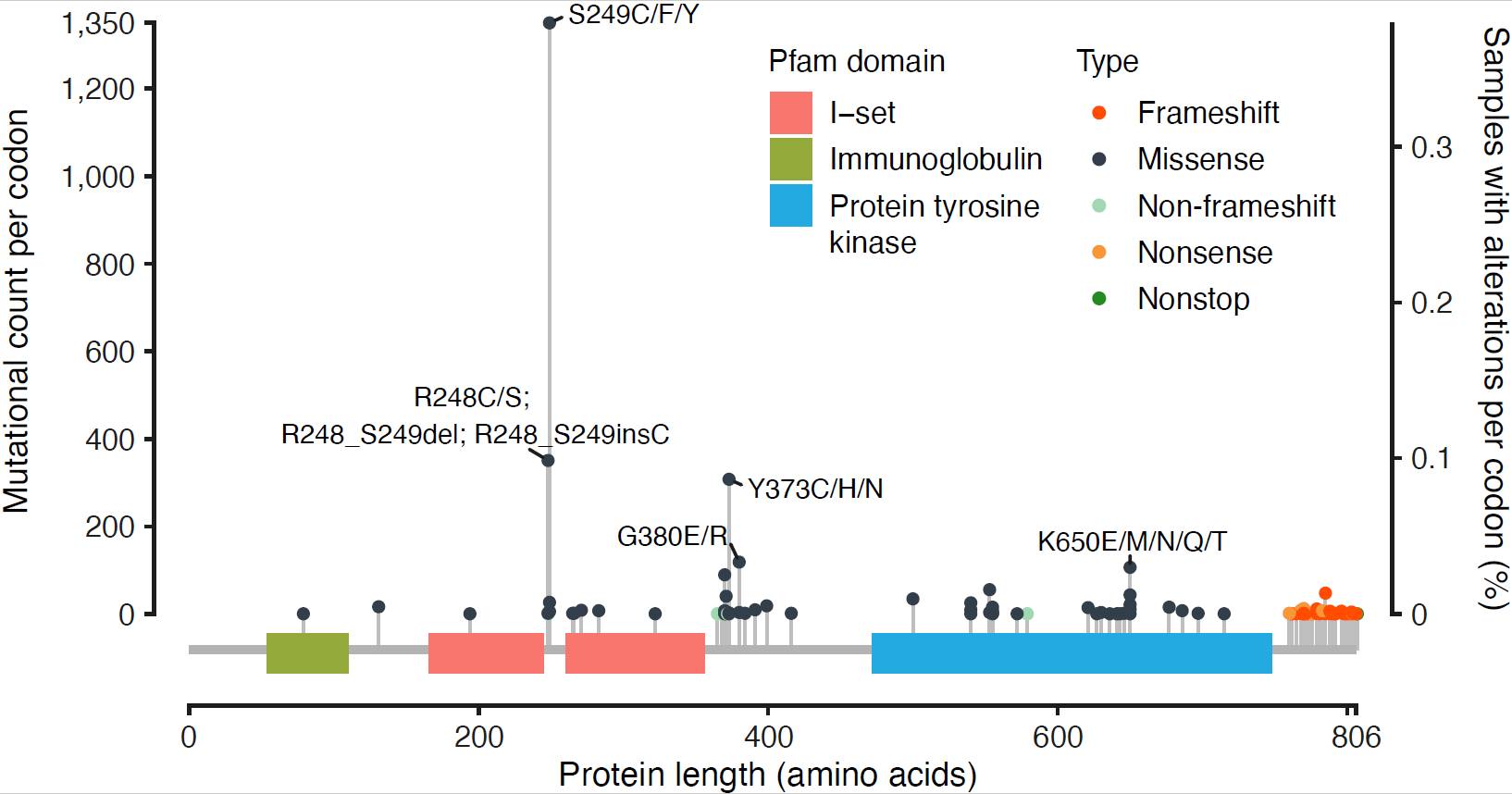


**D**


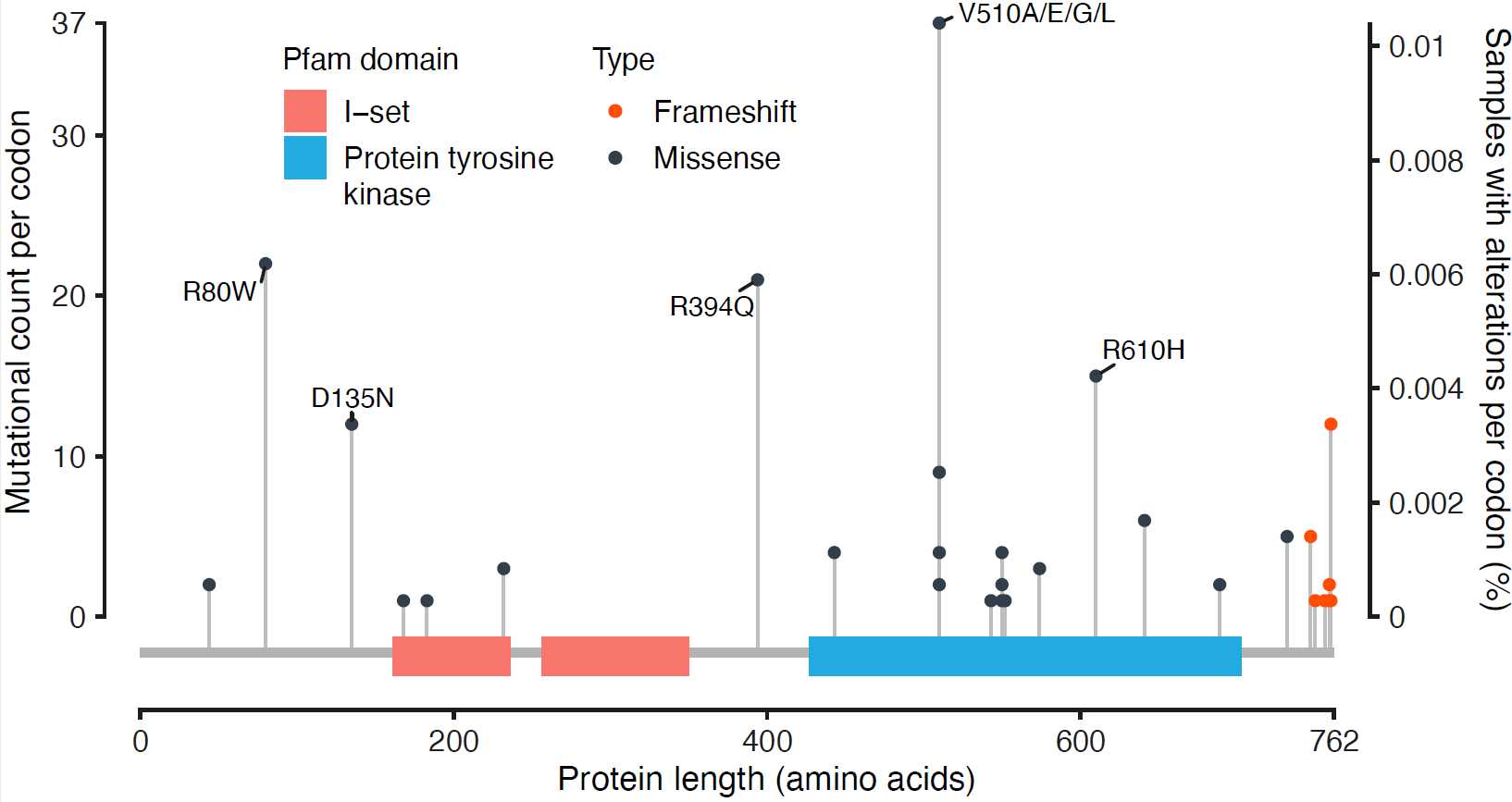


**Supplementary Figure S3. Genomic co-mutational landscape of *FGFR3* SVs in bladder cancer.** *FGFR*, fibroblast growth factor receptor; mut/Mb, mutations/megabase; SV, short variant; TMB, tumor mutational burden.


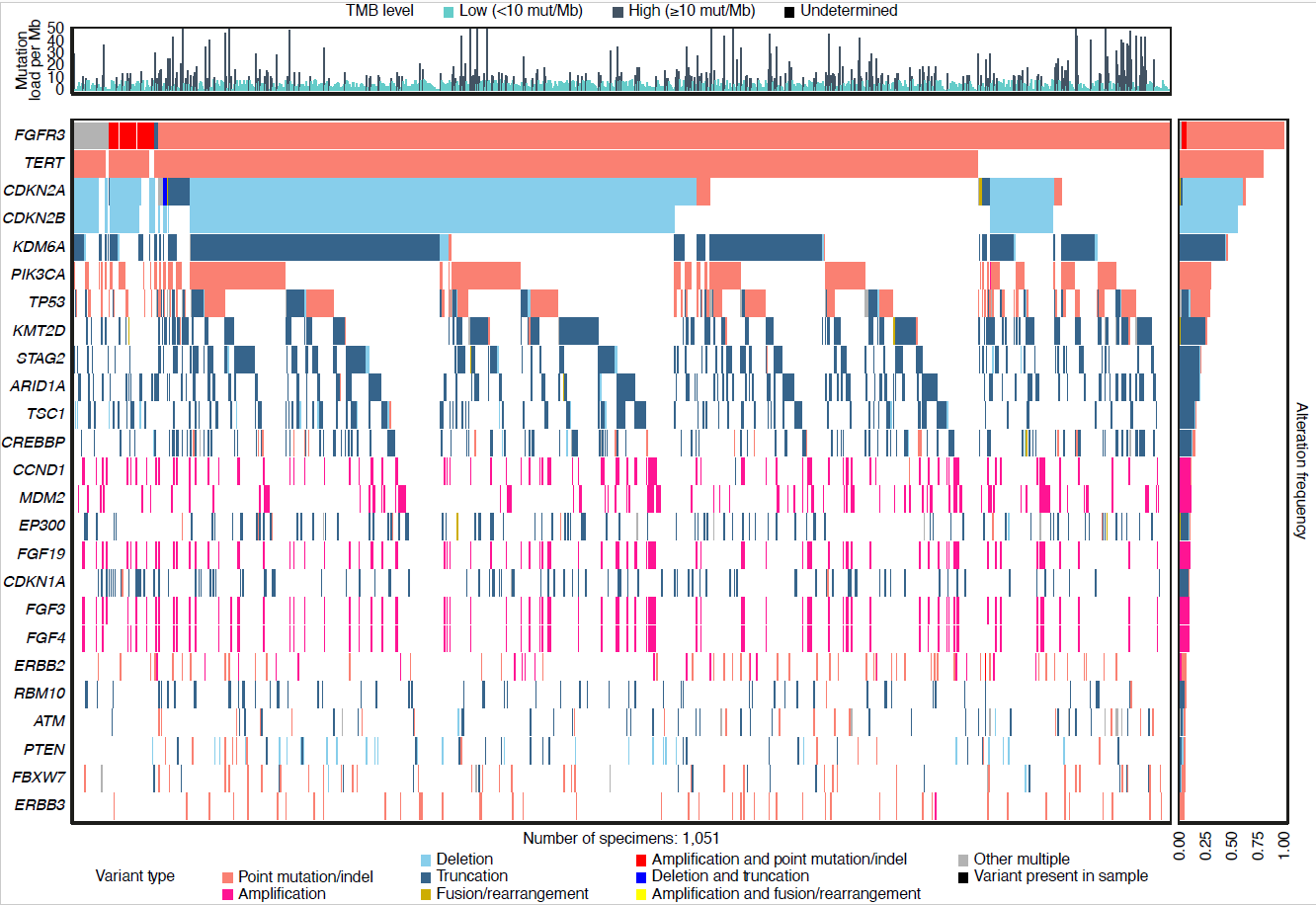


**Supplementary Figure S4. Urinary tract cancer: (A) genomic co-mutational landscape of *FGFR3* SVs and (B) significant co-occurrence and mutual exclusivity of genomic alterations of *FGFR3* SVs.** Orange and blue dots indicate genomic alterations co-occurring and mutually exclusive with *FGFR3* SVs, respectively. The two-tailed Fisher’s exact test was used to estimate the *P* values and odds ratio of associations between genomic alterations and *FGFR3* SVs. The Benjamini–Hochberg procedure was used to estimate the adjusted *P* values. Only genes with a disease prevalence ≥1% were included, and only genes with an adjusted *P* value ≤0.05 were labeled. *FGFR*, fibroblast growth factor receptor; mut/Mb, mutations/megabase; SV, short variant; TMB, tumor mutational burden.

**A**

**
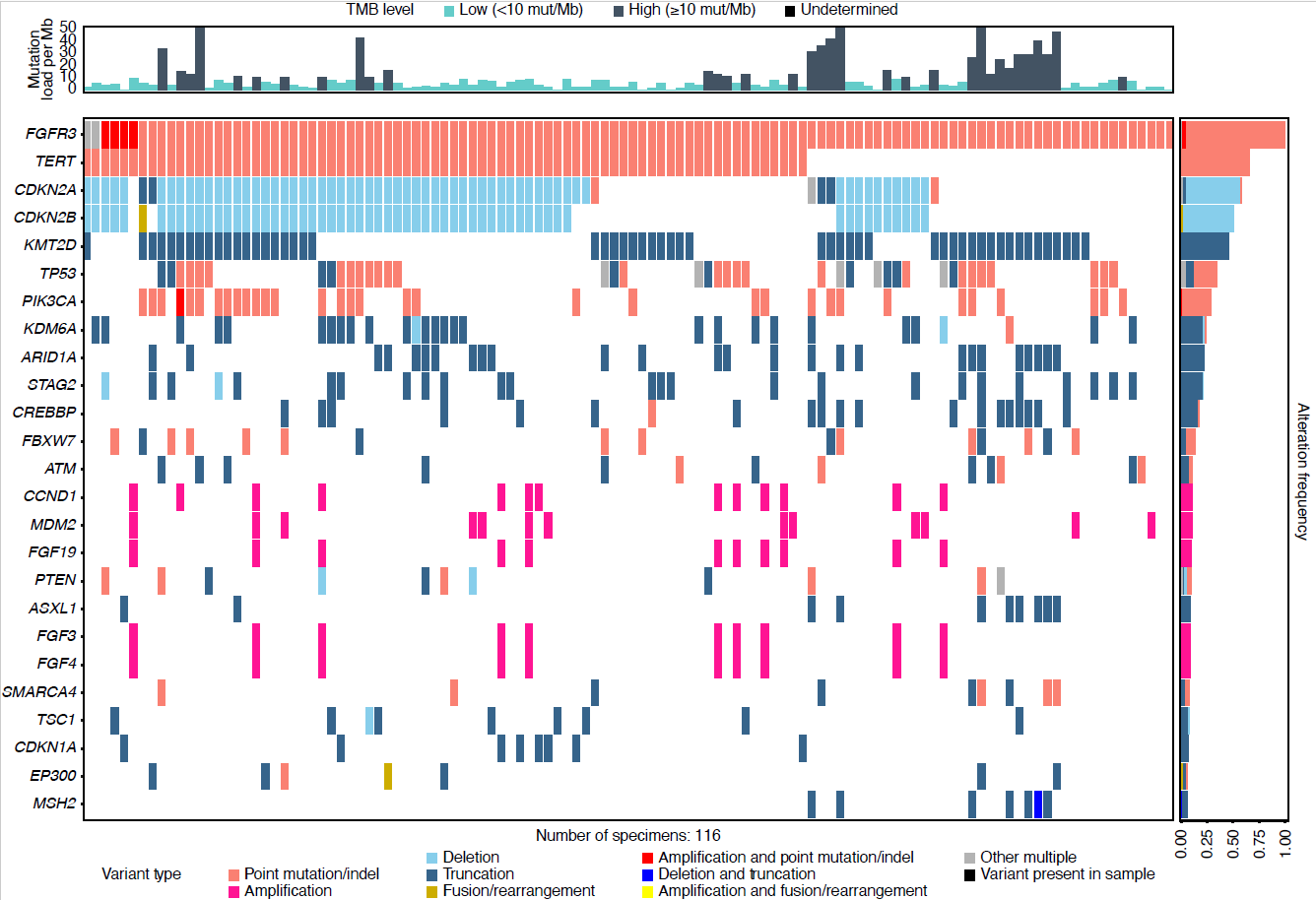
**

**B**

**
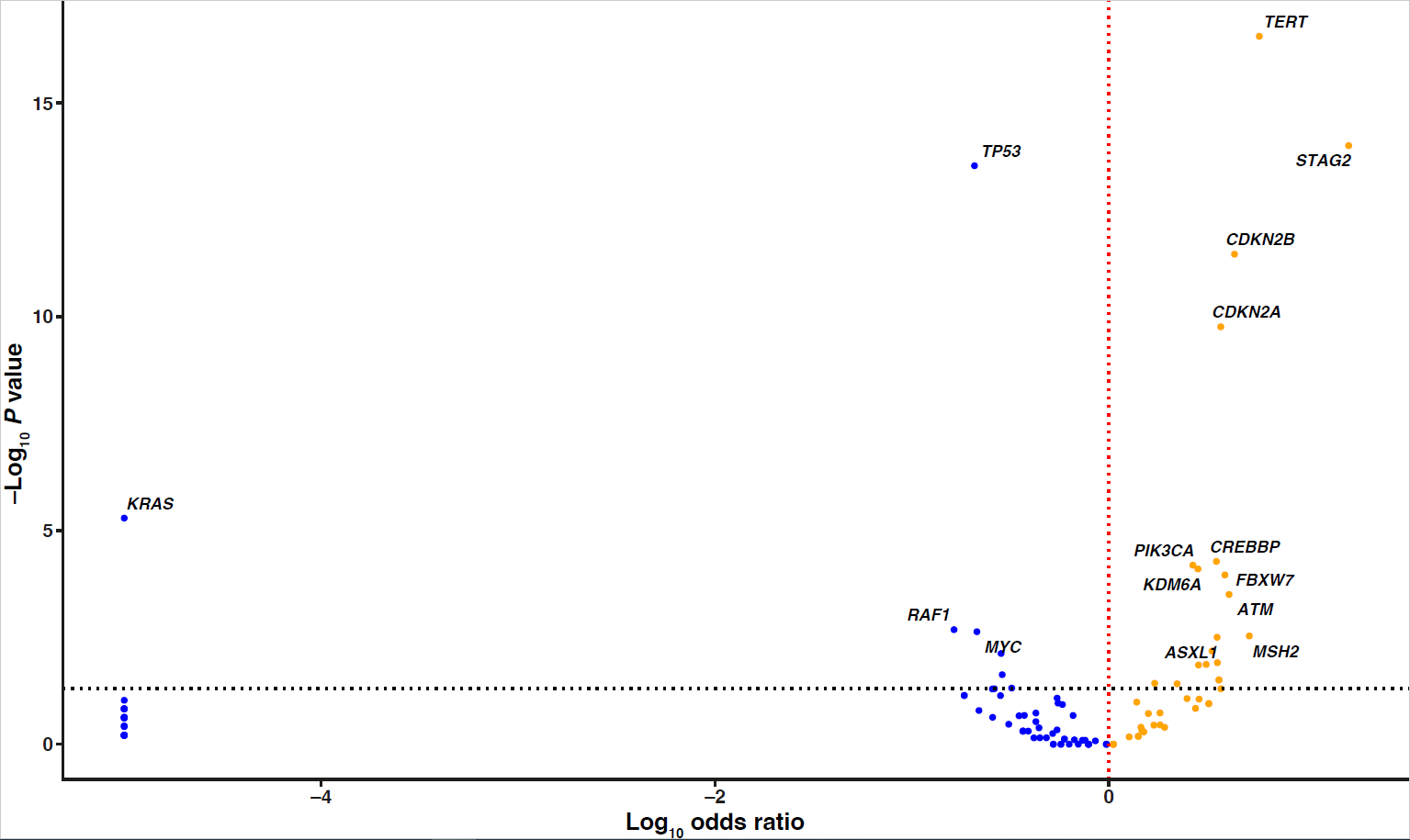
**

**Supplementary Figure S5. Genomic co-mutational landscape of *FGFR2* REs in intra-hepatic cholangiocarcinoma.** *FGFR*, fibroblast growth factor receptor; mut/Mb, mutations/megabase; RE, gene rearrangement; TMB, tumor mutational burden.


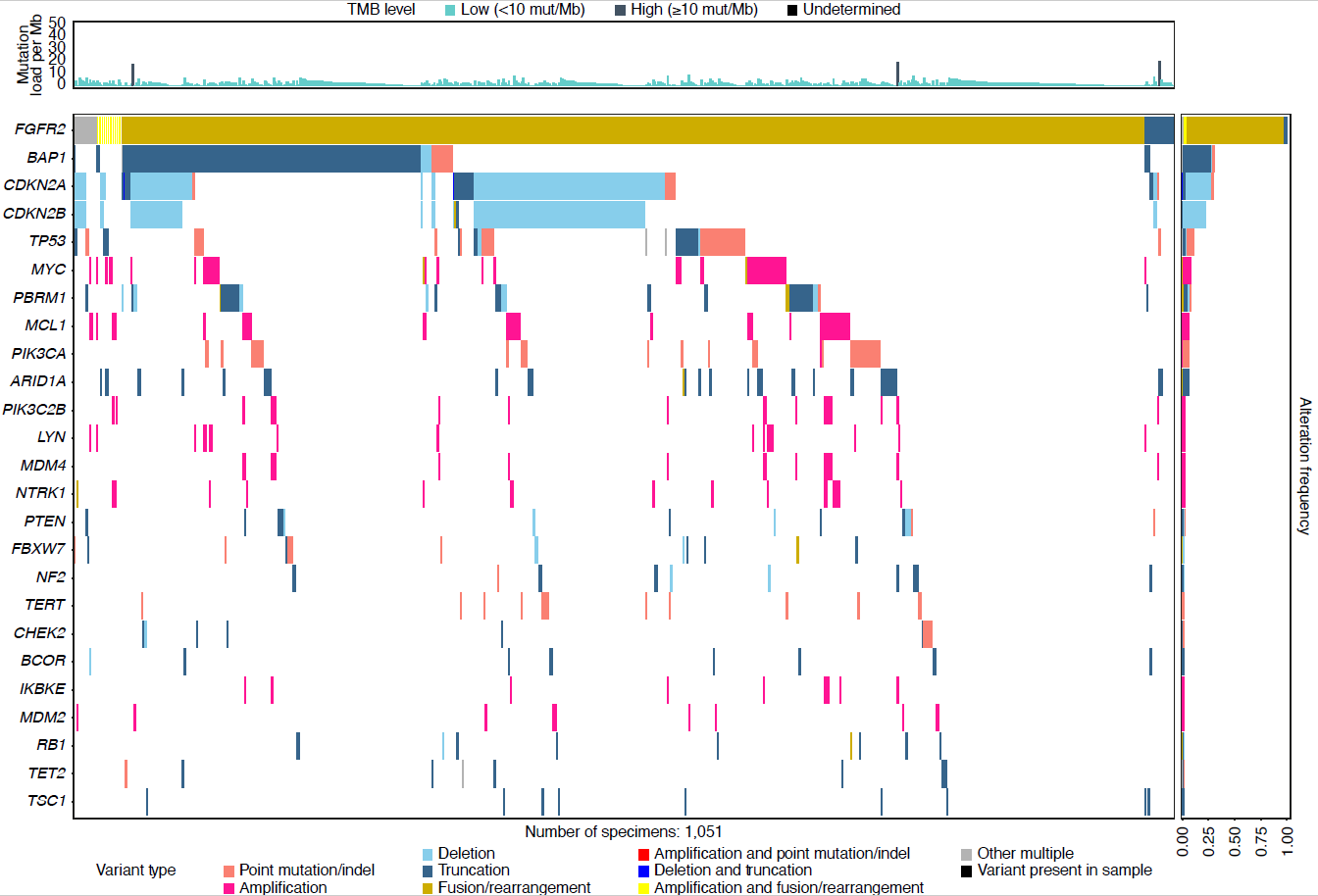


**Supplementary Figure S6. Genomic co-mutational landscape of *FGFR1* SVs in glioma.** *FGFR*, fibroblast growth factor receptor; mut/Mb, mutations/megabase; SV, short variant; TMB, tumor mutational burden.

**
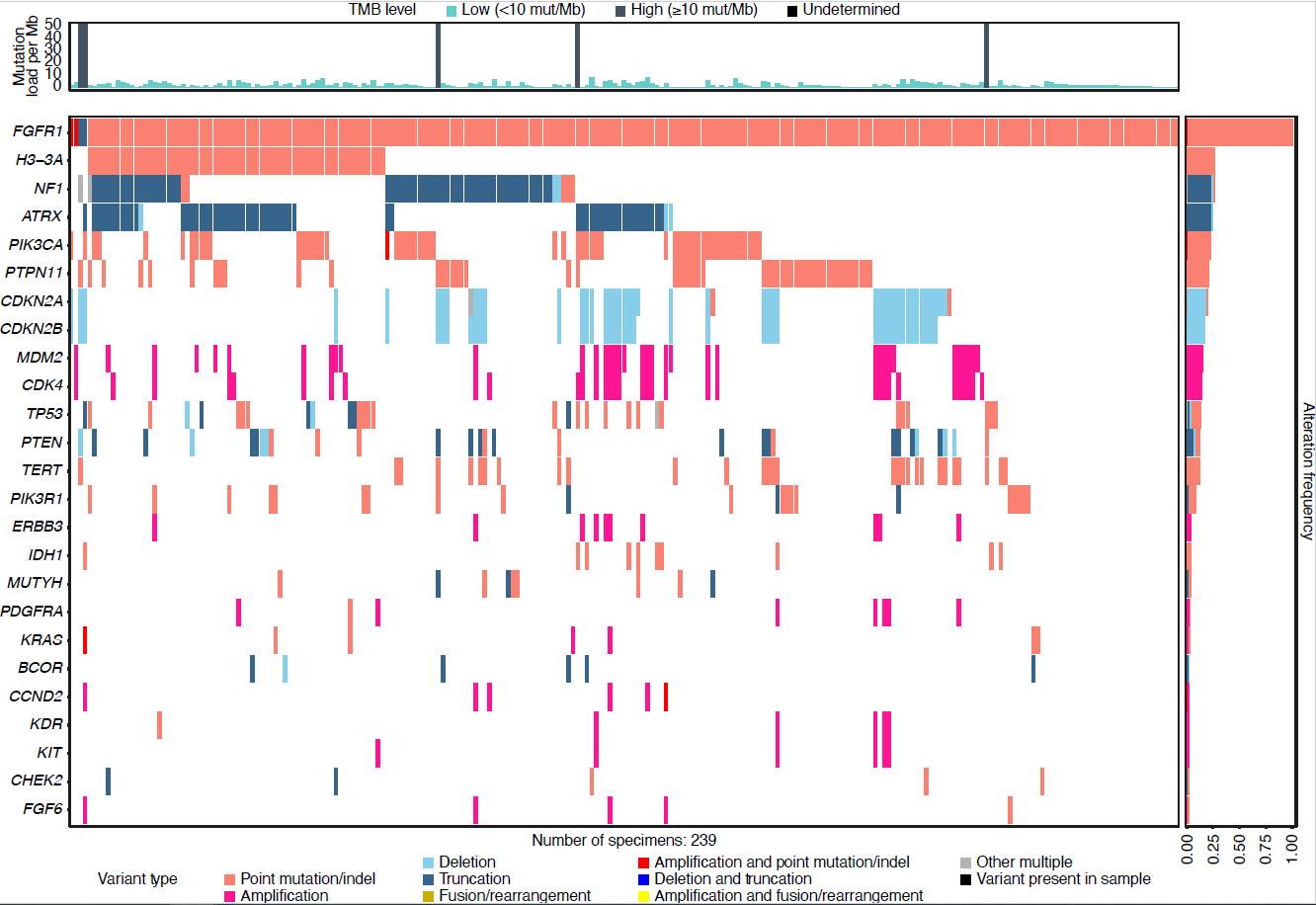
**
