# Supplementary material for: Pan-tumor landscape of fibroblast growth factor receptor 1-4 genomic alterations
Source: ESMO Open. 2022 Nov 30;7(6):100641. doi: 10.1016/j.esmoop.2022.100641 (PMC9832751; doi:10.1016/j.esmoop.2022.100641)
Supplement: Supplementary Tables [file mmc2.docx]

**SUPPLEMENTARY TABLES**

**Supplementary Table S1.** Pan-cancer prevalence of partner genes with *FGFR1-4* REs

| **Partner gene** | **Location** | **Prevalence** | |
| --- | --- | --- | --- |
|  |  | ***n*** | **%** |
| *FGFR1* REs | | | |
| *TACC1* | chr8 | 71 | 25.5 |
| *FGFR1* | chr8 | 50 | 17.9 |
| *PLAG1* | chr8 | 27 | 9.7 |
| *N/A* | chr8 | 18 | 6.5 |
| *BAG4* | chr8 | 16 | 5.7 |
| *LETM2* | chr8 | 7 | 2.5 |
| *ACP3* | chr3 | 4 | 1.4 |
| *N/A* | chr4 | 4 | 1.4 |
| *CP3* | chr3 | 4 | 1.4 |
| *C8orf86* | chr8 | 3 | 1.1 |
| *NSD3* | chr8 | 3 | 1.1 |
| *PLEKHA2* | chr8 | 3 | 1.1 |
| *ADAM9* | chr8 | 2 | 0.7 |
| *IDO2* | chr8 | 2 | 0.7 |
| *MTUS1* | chr8 | 2 | 0.7 |
| *ZNF703* | chr8 | 2 | 0.7 |
| *ADAM18* | chr8 | 1 | 0.4 |
| *ARHGEF18* | chr19 | 1 | 0.4 |
| *ATP6V1H* | chr8 | 1 | 0.4 |
| *BCR* | chr22 | 1 | 0.4 |
| *CGNL1* | chr15 | 1 | 0.4 |
| *CHRNA6* | chr8 | 1 | 0.4 |
| *CNTNAP5* | chr2 | 1 | 0.4 |
| *EFCAB1* | chr8 | 1 | 0.4 |
| *EGFR* | chr7 | 1 | 0.4 |
| *ERC1* | chr12 | 1 | 0.4 |
| *FAM184B* | chr4 | 1 | 0.4 |
| *FLOT1* | chr6 | 1 | 0.4 |
| *FN1* | chr2 | 1 | 0.4 |
| *FOXP1* | chr3 | 1 | 0.4 |
| *FUT10* | chr8 | 1 | 0.4 |
| *G3BP2* | chr4 | 1 | 0.4 |
| *GCOM1;MYZAP* | chr15 | 1 | 0.4 |
| *GFER* | chr16 | 1 | 0.4 |
| *HOMER3* | chr19 | 1 | 0.4 |
| *HOOK3* | chr8 | 1 | 0.4 |
| *HSP90B1* | chr12 | 1 | 0.4 |
| *HTRA4* | chr8 | 1 | 0.4 |
| *IGH* | chr14 | 1 | 0.4 |
| *INSR* | chr19 | 1 | 0.4 |
| *INTS9* | chr8 | 1 | 0.4 |
| *KIF16B* | chr20 | 1 | 0.4 |
| *LHFPL4* | chr3 | 1 | 0.4 |
| *LRRC28* | chr15 | 1 | 0.4 |
| *MAGI2* | chr7 | 1 | 0.4 |
| *MARCH2* | chr19 | 1 | 0.4 |
| *MROH5* | chr8 | 1 | 0.4 |
| *MYO5A* | chr15 | 1 | 0.4 |
| *N/A* | chr11 | 1 | 0.4 |
| *N/A* | chr12 | 1 | 0.4 |
| *N/A* | chr13 | 1 | 0.4 |
| *N/A* | chr2 | 1 | 0.4 |
| *N/A* | chr20 | 1 | 0.4 |
| *N/A* | chr9 | 1 | 0.4 |
| *NBN* | chr8 | 1 | 0.4 |
| *NCOR1* | chr17 | 1 | 0.4 |
| *NRG1* | chr8 | 1 | 0.4 |
| *PALM2* | chr9 | 1 | 0.4 |
| *PCM1* | chr8 | 1 | 0.4 |
| *PLAT* | chr8 | 1 | 0.4 |
| *PLEC* | chr8 | 1 | 0.4 |
| *PLXDC1* | chr17 | 1 | 0.4 |
| *POF1B* | chrX | 1 | 0.4 |
| *PPP2R2A* | chr8 | 1 | 0.4 |
| *PRDM1* | chr6 | 1 | 0.4 |
| *PRKAR2A* | chr3 | 1 | 0.4 |
| *PRKDC* | chr8 | 1 | 0.4 |
| *PXDNL* | chr8 | 1 | 0.4 |
| *RAB11FIP1* | chr8 | 1 | 0.4 |
| *ROBO1* | chr3 | 1 | 0.4 |
| *RRBP1* | chr20 | 1 | 0.4 |
| *SCARA5* | chr8 | 1 | 0.4 |
| *SDCBP* | chr8 | 1 | 0.4 |
| *SNTG1* | chr8 | 1 | 0.4 |
| *SRPRB* | chr3 | 1 | 0.4 |
| *TAFA5* | chr22 | 1 | 0.4 |
| *TG* | chr8 | 1 | 0.4 |
| *UNC5D* | chr8 | 1 | 0.4 |
| *USP25* | chr21 | 1 | 0.4 |
| *WDFY4* | chr10 | 1 | 0.4 |
| *WWP1* | chr8 | 1 | 0.4 |
| *ZMAT4* | chr8 | 1 | 0.4 |
| *FGFR2* REs | | | |
| *N/A* | chr10 | 297 | 16.0 |
| *BICC1* | chr10 | 288 | 15.5 |
| *TACC2* | chr10 | 90 | 4.9 |
| *AHCYL1* | chr1 | 35 | 1.9 |
| *KIAA1217* | chr10 | 32 | 1.7 |
| *SORBS1* | chr10 | 32 | 1.7 |
| *CCDC6* | chr10 | 30 | 1.6 |
| *ATE1* | chr10 | 26 | 1.4 |
| *SHTN1* | chr10 | 26 | 1.4 |
| *WAC* | chr10 | 23 | 1.2 |
| *VCL* | chr10 | 22 | 1.2 |
| *FGFR2* | chr10 | 20 | 1.1 |
| *NRAP* | chr10 | 19 | 1.0 |
| *CTNNA3* | chr10 | 17 | 0.9 |
| *N/A* | chr1 | 17 | 0.9 |
| *N/A* | chr4 | 17 | 0.9 |
| *N/A* | chr12 | 14 | 0.8 |
| *NOL4* | chr18 | 14 | 0.8 |
| *PAWR* | chr12 | 14 | 0.8 |
| *INA* | chr10 | 13 | 0.7 |
| *CASP7* | chr10 | 12 | 0.7 |
| *N/A* | chr6 | 12 | 0.7 |
| *SLMAP* | chr3 | 12 | 0.7 |
| *DDX21* | chr10 | 11 | 0.6 |
| *CIT* | chr12 | 10 | 0.5 |
| *SHROOM3* | chr4 | 10 | 0.5 |
| *POC1B* | chr12 | 9 | 0.5 |
| *TRIM8* | chr10 | 9 | 0.5 |
| *N/A* | chr13 | 8 | 0.4 |
| *N/A* | chr2 | 8 | 0.4 |
| *PAH* | chr12 | 8 | 0.4 |
| *BEND3* | chr6 | 7 | 0.4 |
| *N/A* | chr11 | 7 | 0.4 |
| *N/A* | chr7 | 7 | 0.4 |
| *TTC28* | chr22 | 7 | 0.4 |
| *AFF4* | chr5 | 6 | 0.3 |
| *CCDC186* | chr10 | 6 | 0.3 |
| *ERC1* | chr12 | 6 | 0.3 |
| *FILIP1* | chr6 | 6 | 0.3 |
| *FOXP1* | chr3 | 6 | 0.3 |
| *NRBF2* | chr10 | 6 | 0.3 |
| *BICD1* | chr12 | 5 | 0.3 |
| *BTBD16* | chr10 | 5 | 0.3 |
| *CBX5* | chr12 | 5 | 0.3 |
| *CCAR1* | chr10 | 5 | 0.3 |
| *CFAP57* | chr1 | 5 | 0.3 |
| *DSP* | chr6 | 5 | 0.3 |
| *ETV6* | chr12 | 5 | 0.3 |
| *G3BP2* | chr4 | 5 | 0.3 |
| *KCTD1* | chr18 | 5 | 0.3 |
| *PHLDB2* | chr3 | 5 | 0.3 |
| *PRDX3* | chr10 | 5 | 0.3 |
| *TACC1* | chr8 | 5 | 0.3 |
| *TXLNA* | chr1 | 5 | 0.3 |
| *ZMYM4* | chr1 | 5 | 0.3 |
| *LGSN* | chr6 | 4 | 0.2 |
| *N/A* | chr15 | 4 | 0.2 |
| *N/A* | chr19 | 4 | 0.2 |
| *N/A* | chr3 | 4 | 0.2 |
| *N/A* | chr5 | 4 | 0.2 |
| *N/A* | chrX | 4 | 0.2 |
| *OPTN* | chr10 | 4 | 0.2 |
| *PLPP4* | chr10 | 4 | 0.2 |
| *POF1B* | chrX | 4 | 0.2 |
| *SH3KBP1* | chrX | 4 | 0.2 |
| *ACLY* | chr17 | 3 | 0.2 |
| *AFF2* | chrX | 3 | 0.2 |
| *AHCYL2* | chr7 | 3 | 0.2 |
| *ARHGAP22* | chr10 | 3 | 0.2 |
| *ATF7* | chr12 | 3 | 0.2 |
| *CCDC170* | chr6 | 3 | 0.2 |
| *CELF2* | chr10 | 3 | 0.2 |
| *CFAP58* | chr10 | 3 | 0.2 |
| *DST* | chr6 | 3 | 0.2 |
| *EEA1* | chr12 | 3 | 0.2 |
| *FHL2* | chr2 | 3 | 0.2 |
| *FLACC1* | chr2 | 3 | 0.2 |
| *GAB2* | chr11 | 3 | 0.2 |
| *HTRA1* | chr10 | 3 | 0.2 |
| *IKZF2* | chr2 | 3 | 0.2 |
| *MACF1* | chr1 | 3 | 0.2 |
| *MBIP* | chr14 | 3 | 0.2 |
| *MCU* | chr10 | 3 | 0.2 |
| *MYH15* | chr3 | 3 | 0.2 |
| *N/A* | chr17 | 3 | 0.2 |
| *N/A* | chr8 | 3 | 0.2 |
| *PKD2L1* | chr10 | 3 | 0.2 |
| *RBFOX2* | chr22 | 3 | 0.2 |
| *RBM20* | chr10 | 3 | 0.2 |
| *SH3GLB1* | chr1 | 3 | 0.2 |
| *SORBS2* | chr4 | 3 | 0.2 |
| *SPECC1* | chr17 | 3 | 0.2 |
| *SYNPO2* | chr4 | 3 | 0.2 |
| *TFCP2* | chr12 | 3 | 0.2 |
| *TXLNG* | chrX | 3 | 0.2 |
| *ALDH18A1* | chr10 | 2 | 0.1 |
| *AMOT* | chrX | 2 | 0.1 |
| *ANK3* | chr10 | 2 | 0.1 |
| *ATAD2* | chr8 | 2 | 0.1 |
| *BAIAP2* | chr17 | 2 | 0.1 |
| *BFSP2* | chr3 | 2 | 0.1 |
| *C10orf143* | chr10 | 2 | 0.1 |
| *CAMK2G* | chr10 | 2 | 0.1 |
| *CEP112* | chr17 | 2 | 0.1 |
| *CEP131* | chr17 | 2 | 0.1 |
| *CEP162* | chr6 | 2 | 0.1 |
| *CIP2A* | chr3 | 2 | 0.1 |
| *CLIP1* | chr12 | 2 | 0.1 |
| *COL16A1* | chr1 | 2 | 0.1 |
| *CREB5* | chr7 | 2 | 0.1 |
| *DCDC2* | chr6 | 2 | 0.1 |
| *DIAPH3* | chr13 | 2 | 0.1 |
| *DOCK1* | chr10 | 2 | 0.1 |
| *EBF3* | chr10 | 2 | 0.1 |
| *EVI5* | chr1 | 2 | 0.1 |
| *FLNB* | chr3 | 2 | 0.1 |
| *GAB1* | chr4 | 2 | 0.1 |
| *GKAP1* | chr9 | 2 | 0.1 |
| *HOOK1* | chr1 | 2 | 0.1 |
| *IDE* | chr10 | 2 | 0.1 |
| *INSC* | chr11 | 2 | 0.1 |
| *KCNH7* | chr2 | 2 | 0.1 |
| *KCNMA1* | chr10 | 2 | 0.1 |
| *LRBA* | chr4 | 2 | 0.1 |
| *LRRFIP2* | chr3 | 2 | 0.1 |
| *MATR3* | chr5 | 2 | 0.1 |
| *MCC* | chr5 | 2 | 0.1 |
| *MYLK* | chr3 | 2 | 0.1 |
| *MYOZ1* | chr10 | 2 | 0.1 |
| *N/A* | chr14 | 2 | 0.1 |
| *N/A* | chr18 | 2 | 0.1 |
| *N/A* | chr20 | 2 | 0.1 |
| *N/A* | chr9 | 2 | 0.1 |
| *NARS1* | chr18 | 2 | 0.1 |
| *NPM1* | chr5 | 2 | 0.1 |
| *NRL* | chr14 | 2 | 0.1 |
| *NSMCE4A* | chr10 | 2 | 0.1 |
| *OFD1* | chrX | 2 | 0.1 |
| *PCM1* | chr8 | 2 | 0.1 |
| *PLEKHA1* | chr10 | 2 | 0.1 |
| *PMFBP1* | chr16 | 2 | 0.1 |
| *PPHLN1* | chr12 | 2 | 0.1 |
| *RAB11FIP2* | chr10 | 2 | 0.1 |
| *RABGAP1L* | chr1 | 2 | 0.1 |
| *RAD51* | chr15 | 2 | 0.1 |
| *RBFOX1* | chr16 | 2 | 0.1 |
| *SAFB* | chr19 | 2 | 0.1 |
| *SFMBT2* | chr10 | 2 | 0.1 |
| *SLC28A3* | chr9 | 2 | 0.1 |
| *TAX1BP1* | chr7 | 2 | 0.1 |
| *TIMM23* | chr10 | 2 | 0.1 |
| *TMPO* | chr12 | 2 | 0.1 |
| *TNIP3* | chr4 | 2 | 0.1 |
| *TNS1* | chr2 | 2 | 0.1 |
| *TP73* | chr1 | 2 | 0.1 |
| *TRIM54* | chr2 | 2 | 0.1 |
| *TXLNB* | chr6 | 2 | 0.1 |
| *UACA* | chr15 | 2 | 0.1 |
| *USO1* | chr4 | 2 | 0.1 |
| *USP33* | chr1 | 2 | 0.1 |
| *UTRN* | chr6 | 2 | 0.1 |
| *WDR11* | chr10 | 2 | 0.1 |
| *XPNPEP1* | chr10 | 2 | 0.1 |
| *ZFHX3* | chr16 | 2 | 0.1 |
| *ZMYND11* | chr10 | 2 | 0.1 |
| *ZNF608* | chr5 | 2 | 0.1 |
| *A1CF* | chr10 | 1 | 0.05 |
| *ABI1* | chr10 | 1 | 0.05 |
| *ABLIM1* | chr10 | 1 | 0.05 |
| *ABRAXAS2* | chr10 | 1 | 0.05 |
| *AFF1* | chr4 | 1 | 0.05 |
| *AGAP1* | chr2 | 1 | 0.05 |
| *AIFM1* | chrX | 1 | 0.05 |
| *AKAP7* | chr6 | 1 | 0.05 |
| *AKAP9* | chr7 | 1 | 0.05 |
| *ALCAM* | chr3 | 1 | 0.05 |
| *ALDH1L1* | chr3 | 1 | 0.05 |
| *ALK* | chr2 | 1 | 0.05 |
| *AMBRA1* | chr11 | 1 | 0.05 |
| *AMN1* | chr12 | 1 | 0.05 |
| *ANKRD13C* | chr1 | 1 | 0.05 |
| *ANKRD24* | chr19 | 1 | 0.05 |
| *ANKS1B* | chr12 | 1 | 0.05 |
| *ANXA11* | chr10 | 1 | 0.05 |
| *APIP* | chr11 | 1 | 0.05 |
| *ARHGAP24* | chr4 | 1 | 0.05 |
| *ARID5B* | chr10 | 1 | 0.05 |
| *ASXL3* | chr18 | 1 | 0.05 |
| *ATF1* | chr12 | 1 | 0.05 |
| *ATF2* | chr2 | 1 | 0.05 |
| *ATP10B* | chr5 | 1 | 0.05 |
| *ATRNL1* | chr10 | 1 | 0.05 |
| *AUTS2* | chr7 | 1 | 0.05 |
| *AXDND1* | chr1 | 1 | 0.05 |
| *BAIAP2L1* | chr7 | 1 | 0.05 |
| *BCAS1* | chr20 | 1 | 0.05 |
| *BCCIP;DHX32* | chr10 | 1 | 0.05 |
| *BICD2* | chr9 | 1 | 0.05 |
| *BMPER* | chr7 | 1 | 0.05 |
| *BNIP2* | chr15 | 1 | 0.05 |
| *BTRC* | chr10 | 1 | 0.05 |
| *BZW2* | chr7 | 1 | 0.05 |
| *C1orf50* | chr1 | 1 | 0.05 |
| *CALCOCO1* | chr12 | 1 | 0.05 |
| *CALN1* | chr7 | 1 | 0.05 |
| *CAP2* | chr6 | 1 | 0.05 |
| *CAPRIN1* | chr11 | 1 | 0.05 |
| *CASC2* | chr10 | 1 | 0.05 |
| *CCAR2* | chr8 | 1 | 0.05 |
| *CCDC158* | chr4 | 1 | 0.05 |
| *CCDC18* | chr1 | 1 | 0.05 |
| *CCDC3* | chr10 | 1 | 0.05 |
| *CCDC73* | chr11 | 1 | 0.05 |
| *CCDC88C* | chr14 | 1 | 0.05 |
| *CCM2* | chr7 | 1 | 0.05 |
| *CCSER2* | chr10 | 1 | 0.05 |
| *CD2AP* | chr6 | 1 | 0.05 |
| *CDC42BPA* | chr1 | 1 | 0.05 |
| *CDK5RAP2* | chr9 | 1 | 0.05 |
| *CDYL* | chr6 | 1 | 0.05 |
| *CEBPA* | chr19 | 1 | 0.05 |
| *CEP128* | chr14 | 1 | 0.05 |
| *CEP135* | chr4 | 1 | 0.05 |
| *CEP55* | chr10 | 1 | 0.05 |
| *CEP85* | chr1 | 1 | 0.05 |
| *CGNL1* | chr15 | 1 | 0.05 |
| *CHD9* | chr16 | 1 | 0.05 |
| *CHST15* | chr10 | 1 | 0.05 |
| *CIZ1* | chr9 | 1 | 0.05 |
| *CLMN* | chr14 | 1 | 0.05 |
| *CLOCK* | chr4 | 1 | 0.05 |
| *CMTR2* | chr16 | 1 | 0.05 |
| *COBLL1* | chr2 | 1 | 0.05 |
| *CORIN* | chr4 | 1 | 0.05 |
| *CPSF7* | chr11 | 1 | 0.05 |
| *CREM* | chr10 | 1 | 0.05 |
| *CROCC* | chr1 | 1 | 0.05 |
| *CRYL1* | chr13 | 1 | 0.05 |
| *CTBP2* | chr10 | 1 | 0.05 |
| *CTNNA2* | chr2 | 1 | 0.05 |
| *CTNNB1* | chr3 | 1 | 0.05 |
| *CUBN* | chr10 | 1 | 0.05 |
| *CUL3* | chr2 | 1 | 0.05 |
| *CUZD1* | chr10 | 1 | 0.05 |
| *DAAM2* | chr6 | 1 | 0.05 |
| *DACH2* | chrX | 1 | 0.05 |
| *DBP* | chr19 | 1 | 0.05 |
| *DCP1A* | chr3 | 1 | 0.05 |
| *DEUP1* | chr11 | 1 | 0.05 |
| *DHRSX* | chrX | 1 | 0.05 |
| *DHX9* | chr1 | 1 | 0.05 |
| *DIS3L2* | chr2 | 1 | 0.05 |
| *DMBT1* | chr10 | 1 | 0.05 |
| *DNAJC12* | chr10 | 1 | 0.05 |
| *DNMBP* | chr10 | 1 | 0.05 |
| *DNMT3A* | chr2 | 1 | 0.05 |
| *DZANK1* | chr20 | 1 | 0.05 |
| *DZIP1* | chr13 | 1 | 0.05 |
| *EIF2AK4* | chr15 | 1 | 0.05 |
| *EIF3A* | chr10 | 1 | 0.05 |
| *EMSY* | chr11 | 1 | 0.05 |
| *EPN2* | chr17 | 1 | 0.05 |
| *EPRS1* | chr1 | 1 | 0.05 |
| *ERBB3* | chr12 | 1 | 0.05 |
| *ERICH6B* | chr13 | 1 | 0.05 |
| *EYS* | chr6 | 1 | 0.05 |
| *FAM104A* | chr17 | 1 | 0.05 |
| *FAM120A* | chr9 | 1 | 0.05 |
| *FAM124B* | chr2 | 1 | 0.05 |
| *FAM184B* | chr4 | 1 | 0.05 |
| *FAM240A* | chr3 | 1 | 0.05 |
| *FBXO9* | chr6 | 1 | 0.05 |
| *FHAD1* | chr1 | 1 | 0.05 |
| *FNDC3A* | chr13 | 1 | 0.05 |
| *FSTL1* | chr3 | 1 | 0.05 |
| *FSTL5* | chr4 | 1 | 0.05 |
| *GATA4* | chr8 | 1 | 0.05 |
| *GFRA1* | chr10 | 1 | 0.05 |
| *GMNN* | chr6 | 1 | 0.05 |
| *GPD2* | chr2 | 1 | 0.05 |
| *GPHN* | chr14 | 1 | 0.05 |
| *GPX3* | chr5 | 1 | 0.05 |
| *GRHL2* | chr8 | 1 | 0.05 |
| *GRK5* | chr10 | 1 | 0.05 |
| *GRSF1* | chr4 | 1 | 0.05 |
| *HDAC8* | chrX | 1 | 0.05 |
| *HECW1* | chr7 | 1 | 0.05 |
| *HIP1* | chr7 | 1 | 0.05 |
| *HK1* | chr10 | 1 | 0.05 |
| *HMGCLL1* | chr6 | 1 | 0.05 |
| *HNRNPA1* | chr12 | 1 | 0.05 |
| *HPGDS* | chr4 | 1 | 0.05 |
| *HSPA12A* | chr10 | 1 | 0.05 |
| *IFI35* | chr17 | 1 | 0.05 |
| *IQCG* | chr3 | 1 | 0.05 |
| *IQGAP2* | chr5 | 1 | 0.05 |
| *ITGA8* | chr10 | 1 | 0.05 |
| *ITGB1* | chr10 | 1 | 0.05 |
| *ITIH2* | chr10 | 1 | 0.05 |
| *ITIH5* | chr10 | 1 | 0.05 |
| *ITPR1* | chr3 | 1 | 0.05 |
| *JMJD1C* | chr10 | 1 | 0.05 |
| *KCNH1* | chr1 | 1 | 0.05 |
| *KCNN3* | chr1 | 1 | 0.05 |
| *KCTD18* | chr2 | 1 | 0.05 |
| *KCTD3* | chr1 | 1 | 0.05 |
| *KDM1B* | chr6 | 1 | 0.05 |
| *KHDRBS1* | chr1 | 1 | 0.05 |
| *KIDINS220* | chr2 | 1 | 0.05 |
| *KIF5A* | chr12 | 1 | 0.05 |
| *KIF5C* | chr2 | 1 | 0.05 |
| *KIF7* | chr15 | 1 | 0.05 |
| *KPNA1* | chr3 | 1 | 0.05 |
| *KRT4* | chr12 | 1 | 0.05 |
| *KRT7* | chr12 | 1 | 0.05 |
| *KRT80* | chr12 | 1 | 0.05 |
| *KTN1* | chr14 | 1 | 0.05 |
| *L3MBTL3* | chr6 | 1 | 0.05 |
| *LARP6* | chr15 | 1 | 0.05 |
| *LCORL* | chr4 | 1 | 0.05 |
| *LINC00359* | chr13 | 1 | 0.05 |
| *LIPJ* | chr10 | 1 | 0.05 |
| *LIX1* | chr5 | 1 | 0.05 |
| *LPP* | chr3 | 1 | 0.05 |
| *LPXN* | chr11 | 1 | 0.05 |
| *LRMDA* | chr10 | 1 | 0.05 |
| *LUZP4* | chrX | 1 | 0.05 |
| *LY75* | chr2 | 1 | 0.05 |
| *LYRM4* | chr6 | 1 | 0.05 |
| *LZTFL1* | chr3 | 1 | 0.05 |
| *LZTS2* | chr10 | 1 | 0.05 |
| *MAGI3* | chr1 | 1 | 0.05 |
| *MAP3K1* | chr5 | 1 | 0.05 |
| *MAP3K5* | chr6 | 1 | 0.05 |
| *MLEC* | chr12 | 1 | 0.05 |
| *MOB2* | chr11 | 1 | 0.05 |
| *MORC4* | chrX | 1 | 0.05 |
| *MSI2* | chr17 | 1 | 0.05 |
| *MST4* | chrX | 1 | 0.05 |
| *MTMR3* | chr22 | 1 | 0.05 |
| *MYC* | chr8 | 1 | 0.05 |
| *MYH9* | chr22 | 1 | 0.05 |
| *MYO1E* | chr15 | 1 | 0.05 |
| *MYO5B* | chr18 | 1 | 0.05 |
| *MYPN* | chr10 | 1 | 0.05 |
| *MZT1* | chr13 | 1 | 0.05 |
| *N/A* | chr16 | 1 | 0.05 |
| *N/A* | chr21 | 1 | 0.05 |
| *N/A* | chr22 | 1 | 0.05 |
| *NAV3* | chr12 | 1 | 0.05 |
| *NBEA* | chr13 | 1 | 0.05 |
| *NDUFA13* | chr19 | 1 | 0.05 |
| *NEK1* | chr4 | 1 | 0.05 |
| *NHLRC3* | chr13 | 1 | 0.05 |
| *NMI* | chr2 | 1 | 0.05 |
| *NOTCH2* | chr1 | 1 | 0.05 |
| *NSMCE2* | chr8 | 1 | 0.05 |
| *ODAD4* | chr17 | 1 | 0.05 |
| *OGA* | chr10 | 1 | 0.05 |
| *ORC6* | chr16 | 1 | 0.05 |
| *PAPPA2* | chr1 | 1 | 0.05 |
| *PCSK5* | chr9 | 1 | 0.05 |
| *PCSK7* | chr11 | 1 | 0.05 |
| *PDE2A* | chr11 | 1 | 0.05 |
| *PDE3B* | chr11 | 1 | 0.05 |
| *PDHX* | chr11 | 1 | 0.05 |
| *PDZRN3* | chr3 | 1 | 0.05 |
| *PDZRN4* | chr12 | 1 | 0.05 |
| *PELP1* | chr17 | 1 | 0.05 |
| *PEX5L* | chr3 | 1 | 0.05 |
| *PFDN2* | chr1 | 1 | 0.05 |
| *PHC1* | chr12 | 1 | 0.05 |
| *PHGDH* | chr1 | 1 | 0.05 |
| *PITRM1* | chr10 | 1 | 0.05 |
| *PKP4* | chr2 | 1 | 0.05 |
| *PLCE1* | chr10 | 1 | 0.05 |
| *PLEC* | chr8 | 1 | 0.05 |
| *PNLIPRP2* | chr10 | 1 | 0.05 |
| *POC1A* | chr3 | 1 | 0.05 |
| *POLDIP3* | chr22 | 1 | 0.05 |
| *POU6F2* | chr7 | 1 | 0.05 |
| *PPIF* | chr10 | 1 | 0.05 |
| *PPP1R21* | chr2 | 1 | 0.05 |
| *PQBP1* | chrX | 1 | 0.05 |
| *PRDM16* | chr1 | 1 | 0.05 |
| *PRKG1* | chr10 | 1 | 0.05 |
| *PRRC2A* | chr6 | 1 | 0.05 |
| *PRXL2A* | chr10 | 1 | 0.05 |
| *PSPC1* | chr13 | 1 | 0.05 |
| *PTBP3* | chr9 | 1 | 0.05 |
| *PTEN* | chr10 | 1 | 0.05 |
| *PXN* | chr12 | 1 | 0.05 |
| *RALGAPA2* | chr20 | 1 | 0.05 |
| *RALY* | chr20 | 1 | 0.05 |
| *RASAL2* | chr1 | 1 | 0.05 |
| *RASGEF1B* | chr4 | 1 | 0.05 |
| *RASGRF1* | chr15 | 1 | 0.05 |
| *RBMS3* | chr3 | 1 | 0.05 |
| *RCHY1* | chr4 | 1 | 0.05 |
| *REXO1* | chr19 | 1 | 0.05 |
| *RGS10* | chr10 | 1 | 0.05 |
| *RIT2* | chr18 | 1 | 0.05 |
| *RNF13* | chr3 | 1 | 0.05 |
| *RNF41* | chr12 | 1 | 0.05 |
| *ROPN1* | chr3 | 1 | 0.05 |
| *RPAP3* | chr12 | 1 | 0.05 |
| *RPTOR* | chr17 | 1 | 0.05 |
| *RTN4IP1* | chr6 | 1 | 0.05 |
| *RUFY1* | chr5 | 1 | 0.05 |
| *RUNX1T1* | chr8 | 1 | 0.05 |
| *SCLT1* | chr4 | 1 | 0.05 |
| *SDC4* | chr20 | 1 | 0.05 |
| *SDCBP* | chr8 | 1 | 0.05 |
| *SEC23IP* | chr10 | 1 | 0.05 |
| *SEL1L3* | chr4 | 1 | 0.05 |
| *SEPT10* | chr2 | 1 | 0.05 |
| *SEPT14* | chr7 | 1 | 0.05 |
| *SEPT8* | chr5 | 1 | 0.05 |
| *SGMS2* | chr4 | 1 | 0.05 |
| *SKI* | chr1 | 1 | 0.05 |
| *SLC35F3* | chr1 | 1 | 0.05 |
| *SLC45A3* | chr1 | 1 | 0.05 |
| *SNRNP200* | chr2 | 1 | 0.05 |
| *SNX1* | chr15 | 1 | 0.05 |
| *SOGA1* | chr20 | 1 | 0.05 |
| *SORBS3* | chr8 | 1 | 0.05 |
| *SORCS1* | chr10 | 1 | 0.05 |
| *SPICE1* | chr3 | 1 | 0.05 |
| *SPOCK3* | chr4 | 1 | 0.05 |
| *SRCIN1* | chr17 | 1 | 0.05 |
| *SREBF2* | chr22 | 1 | 0.05 |
| *ST18* | chr8 | 1 | 0.05 |
| *ST8SIA6* | chr10 | 1 | 0.05 |
| *STAB2* | chr12 | 1 | 0.05 |
| *STAT4* | chr2 | 1 | 0.05 |
| *STK3* | chr8 | 1 | 0.05 |
| *STK4* | chr20 | 1 | 0.05 |
| *STMP1* | chr7 | 1 | 0.05 |
| *SUFU* | chr10 | 1 | 0.05 |
| *SYCP1* | chr1 | 1 | 0.05 |
| *TBATA* | chr10 | 1 | 0.05 |
| *TBC1D1* | chr4 | 1 | 0.05 |
| *TBC1D4* | chr13 | 1 | 0.05 |
| *TBC1D5* | chr3 | 1 | 0.05 |
| *TCERG1L* | chr10 | 1 | 0.05 |
| *TCF7L2* | chr10 | 1 | 0.05 |
| *TENC1;LOC283335* | chr12 | 1 | 0.05 |
| *THAP10* | chr15 | 1 | 0.05 |
| *TIFA* | chr4 | 1 | 0.05 |
| *TJAP1* | chr6 | 1 | 0.05 |
| *TLK1* | chr2 | 1 | 0.05 |
| *TMEM65* | chr8 | 1 | 0.05 |
| *TMF1* | chr3 | 1 | 0.05 |
| *TMIGD1* | chr17 | 1 | 0.05 |
| *TNIP1* | chr5 | 1 | 0.05 |
| *TNKS2* | chr10 | 1 | 0.05 |
| *TNPO3* | chr7 | 1 | 0.05 |
| *TNS3* | chr7 | 1 | 0.05 |
| *TPM1* | chr15 | 1 | 0.05 |
| *TPM4* | chr19 | 1 | 0.05 |
| *TRA2B* | chr3 | 1 | 0.05 |
| *TRAF6* | chr11 | 1 | 0.05 |
| *TRIP11* | chr14 | 1 | 0.05 |
| *TRPC3* | chr4 | 1 | 0.05 |
| *TSC22D1* | chr13 | 1 | 0.05 |
| *TSC22D4* | chr7 | 1 | 0.05 |
| *UBLCP1* | chr5 | 1 | 0.05 |
| *UBQLN1* | chr9 | 1 | 0.05 |
| *UPF2* | chr10 | 1 | 0.05 |
| *USP12* | chr13 | 1 | 0.05 |
| *USP54* | chr10 | 1 | 0.05 |
| *UST* | chr6 | 1 | 0.05 |
| *VWA8* | chr13 | 1 | 0.05 |
| *WBP1L* | chr10 | 1 | 0.05 |
| *WDHD1* | chr14 | 1 | 0.05 |
| *WDR72* | chr15 | 1 | 0.05 |
| *ZMAT3* | chr3 | 1 | 0.05 |
| *ZMYM6* | chr1 | 1 | 0.05 |
| *ZMYND8* | chr20 | 1 | 0.05 |
| *ZNF281* | chr1 | 1 | 0.05 |
| *ZNF318* | chr6 | 1 | 0.05 |
| *ZNF326* | chr1 | 1 | 0.05 |
| *ZNF839* | chr14 | 1 | 0.05 |
| *ZNF92* | chr7 | 1 | 0.05 |
| *ZSCAN4* | chr19 | 1 | 0.05 |
| *FGFR3* REs | | | |
| *TACC3* | chr4 | 954 | 82.4 |
| *N/A* | chr4 | 44 | 3.8 |
| *TNIP2* | chr4 | 15 | 1.3 |
| *LETM1* | chr4 | 11 | 0.9 |
| *NSD2* | chr4 | 10 | 0.9 |
| *FAM53A* | chr4 | 7 | 0.6 |
| *ADD1* | chr4 | 6 | 0.5 |
| *JAKMIP1* | chr4 | 6 | 0.5 |
| *MAEA* | chr4 | 4 | 0.3 |
| *TBC1D1* | chr4 | 4 | 0.3 |
| *N/A* | chr19 | 3 | 0.3 |
| *SLBP* | chr4 | 3 | 0.3 |
| *CLIP2* | chr7 | 2 | 0.2 |
| *DMPK* | chr19 | 2 | 0.2 |
| *DNMT3A* | chr2 | 2 | 0.2 |
| *FAM184B* | chr4 | 2 | 0.2 |
| *MRFAP1L1* | chr4 | 2 | 0.2 |
| *N/A* | chr1 | 2 | 0.2 |
| *NELFA* | chr4 | 2 | 0.2 |
| *PAICS* | chr4 | 2 | 0.2 |
| *PHLDB3* | chr19 | 2 | 0.2 |
| *RGS12* | chr4 | 2 | 0.2 |
| *TMEM129* | chr4 | 2 | 0.2 |
| *ACACA* | chr17 | 1 | 0.09 |
| *ADORA1* | chr1 | 1 | 0.09 |
| *AFF4* | chr5 | 1 | 0.09 |
| *AKAP8L* | chr19 | 1 | 0.09 |
| *AMBRA1* | chr11 | 1 | 0.09 |
| *BEND6* | chr6 | 1 | 0.09 |
| *C4orf19* | chr4 | 1 | 0.09 |
| *CC2D2A* | chr4 | 1 | 0.09 |
| *CCDC149* | chr4 | 1 | 0.09 |
| *CDYL2* | chr16 | 1 | 0.09 |
| *CEP135* | chr4 | 1 | 0.09 |
| *COG4* | chr16 | 1 | 0.09 |
| *COL4A2* | chr13 | 1 | 0.09 |
| *DCTN2* | chr12 | 1 | 0.09 |
| *DNAH17* | chr17 | 1 | 0.09 |
| *FGFR3* | chr4 | 1 | 0.09 |
| *FLJ30838* | chr2 | 1 | 0.09 |
| *FLNA* | chrX | 1 | 0.09 |
| *FRYL* | chr4 | 1 | 0.09 |
| *GAB3* | chrX | 1 | 0.09 |
| *GNAT2* | chr1 | 1 | 0.09 |
| *HMGN2* | chr1 | 1 | 0.09 |
| *HSP90AA1* | chr14 | 1 | 0.09 |
| *IRF2BPL* | chr14 | 1 | 0.09 |
| *KIF1B* | chr1 | 1 | 0.09 |
| *KPNA7* | chr7 | 1 | 0.09 |
| *LAP3* | chr4 | 1 | 0.09 |
| *LIN9* | chr1 | 1 | 0.09 |
| *LMNB2* | chr19 | 1 | 0.09 |
| *MACROD2* | chr20 | 1 | 0.09 |
| *MYH14* | chr19 | 1 | 0.09 |
| *MYO18A* | chr17 | 1 | 0.09 |
| *N/A* | chr11 | 1 | 0.09 |
| *N/A* | chr12 | 1 | 0.09 |
| *N/A* | chr14 | 1 | 0.09 |
| *N/A* | chr15 | 1 | 0.09 |
| *N/A* | chr16 | 1 | 0.09 |
| *N/A* | chr17 | 1 | 0.09 |
| *N/A* | chr18 | 1 | 0.09 |
| *N/A* | chr2 | 1 | 0.09 |
| *N/A* | chr20 | 1 | 0.09 |
| *N/A* | chr22 | 1 | 0.09 |
| *NBR1* | chr17 | 1 | 0.09 |
| *PAPSS1* | chr4 | 1 | 0.09 |
| *PBXIP1* | chr1 | 1 | 0.09 |
| *PGD* | chr1 | 1 | 0.09 |
| *PLCB3* | chr11 | 1 | 0.09 |
| *POLN* | chr4 | 1 | 0.09 |
| *PPL* | chr16 | 1 | 0.09 |
| *PSMC3IP* | chr17 | 1 | 0.09 |
| *RAB6B* | chr3 | 1 | 0.09 |
| *RNF13* | chr3 | 1 | 0.09 |
| *RSRC1* | chr3 | 1 | 0.09 |
| *SAMD8* | chr10 | 1 | 0.09 |
| *SEC23IP* | chr10 | 1 | 0.09 |
| *SKI* | chr1 | 1 | 0.09 |
| *SLC25A25* | chr9 | 1 | 0.09 |
| *SMIM14* | chr4 | 1 | 0.09 |
| *SNX1* | chr15 | 1 | 0.09 |
| *SORCS2* | chr4 | 1 | 0.09 |
| *TBC1D14* | chr4 | 1 | 0.09 |
| *TNIK* | chr3 | 1 | 0.09 |
| *TP53* | chr17 | 1 | 0.09 |
| *TRAF2* | chr9 | 1 | 0.09 |
| *TSPAN7* | chrX | 1 | 0.09 |
| *USO1* | chr4 | 1 | 0.09 |
| *UVSSA* | chr4 | 1 | 0.09 |
| *ZC3H7B* | chr22 | 1 | 0.09 |
| *ZNF326* | chr1 | 1 | 0.09 |
| *ZNF876P* | chr4 | 1 | 0.09 |
| *FGFR4* REs | | | |
| *NSD1* | chr5 | 5 | 45.5 |
| *ANO3* | chr11 | 1 | 9.1 |
| *N/A* | chr5 | 2 | 18.2 |
| *CHD4* | chr12 | 1 | 9.1 |
| *DKK3* | chr11 | 1 | 9.1 |
| *ZNF346* | chr5 | 1 | 9.1 |

N/A denotes an *FGFR* intergenic rearrangement.

chr, chromosome; FGFR, fibroblast growth factor receptor; RE, gene rearrangement.

**Supplementary Table S2.** Comparison of baseline demographics between the *FGFR1-4*-altered and *FGFR1-4*-unaltered pan-cancer cohort

| **Baseline demographic** | ***FGFR1-4*-altered cohort**  **(*n* = 9,603)** | ***FGFR1-4*-unaltered cohort**  **(*n* = 346,210)** | ***P* value^a^** |
| --- | --- | --- | --- |
| Sex, *n* (%)  Male  Female  Unknown | 4,114 (42.8)  5,485 (57.1)  4 (0.04) | 156,367 (45.2)  189,670 (54.8)  173 (0.05) | 6.1x10^-6^ |
| Age^b^  Median (IQR), years  ≥18 years, *n* (%) | *n* = 9,596  65.0 (56.0-73.0)  9,462 (98.6) | *n* = 345,816  64.0 (55.0-72.0)  343,043 (99.1) | 2.6x10^-16^  5.1x10^-9^ |
| Genomic ancestry, *n* (%)  African  Admixed American  East Asian  European  South Asian | 800 (8.3)  825 (8.6)  323 (3.4)  7,563 (78.7)  92 (1.0) | 35,077 (10.1)  28,953 (8.4)  12,640 (3.7)  266,147 (76.8)  3,393 (1.0) | 3.3x10^-9^  0.4  0.1  1.4x10^-5^  0.9 |
| Metastatic,^c^ *n* (%)  Yes | *n* = 7,666  2,961 (38.6) | *n* = 282,631  127,292 (45.0) | 4.2x10^-29^ |

FGFR, fibroblast growth factor receptor; IQR, interquartile range.

^a^*P* values of categorical variables were estimated by the Fisher’s exact test and the comparison of median age was performed using the Wilcoxon rank-sum test.

^b^‘Age’ had a missingness rate of 0.1% in the dataset.

^c^‘Metastatic’ had a missingness rate of 18.4% in the dataset.

**Supplementary Table S3.** Comparison of baseline demographics for specific *FGFR* alterations in particular cancer indications

|  | **Bladder cancer** | | | **Intrahepatic cholangiocarcinoma** | | | **Glioma** | | |
| --- | --- | --- | --- | --- | --- | --- | --- | --- | --- |
| **Baseline demographic** | *FGFR3* SV-altered (*n* = 1,051) | *FGFR3* SV-unaltered  (*n* = 6,688) | *P* value^a^ | *FGFR2* RE-altered (*n* = 618) | *FGFR2*  RE-unaltered  (*n* = 6,023) | *P* value^a^ | *FGFR1* SV-altered (*n* = 239) | *FGFR1*  SV-unaltered  (*n* = 11,311) | *P* value^a^ |
| Sex, *n* (%)  Male  Female  Unknown | 295 (28.1)  756 (71.9)  0 | 1,774 (26.5)  4,911 (73.4)  3 (0.04) | 0.29 | 388 (62.8)  230 (37.2)  0 | 2,992 (49.7)  3,027 (50.3)  4 (0.07) | 4.6x10^-10^ | 113 (47.3)  125 (52.3)  1 (0.4) | 4,746 (42.0)  6,564 (58.0)  1 (0.009) | 0.08 |
| Age  Median (IQR), years  ≥18 years, *n* (%) | 71 (63-78)  0 | 69 (62-76)  1 (0.01) | 4.5x10^-5^  1 | 59 (50-67)  0 | 65 (57-72)  4 (0.07) | <2.3x10^-16^  1 | 30 (16-48.5)  67 (28.0) | 53 (36-63)  1,017 (9.0) | <2.2x10^-16^  6.9x10^-17^ |
| Genomic ancestry, *n* (%)  African  Admixed American  East Asian  European  South Asian | 62 (5.9)  69 (6.6)  20 (1.9)  897 (85.3)  3 (0.3) | 488 (7.3)  358 (5.4)  155 (2.3)  5,626 (84.1)  61 (0.9) | 0.11  0.11  0.50  0.33  0.04 | 69 (11.2)  53 (8.6)  30 (4.9)  460 (74.4)  6 (1.0) | 443 (7.4)  706 (11.7)  299 (5.0)  4,506 (74.8)  69 (1.1) | 0.001  0.02  1  0.84  0.84 | 14 (5.9)  41 (17.2)  8 (3.3)  169 (70.7)  7 (2.9) | 591 (5.2)  1,259 (11.1)  287 (2.5)  8,950 (79.1)  224 (2.0) | 0.66  0.007  0.40  0.002  0.34 |
| Tumor origin, *n* (%)  Local  Metastatic  Unknown | 614 (58.4)  369 (35.1)  68 (6.5) | 3,990 (59.7)  2,123 (31.7)  575 (8.6) | 0.46 | 539 (87.2)  56 (9.1)  23 (3.7) | 4,531 (75.2)  763 (12.7)  729 (12.1) | 1.7x10^-12^ | 218 (91.2)  3 (1.3)  18 (7.5) | 11,088 (98.0)  37 (0.3)  186 (1.6) | 3.3x10^-8^ |

FGFR, fibroblast growth factor receptor; IQR, interquartile range; RE, gene rearrangement; SV, short variant.

^a^*P* values of categorical variables were estimated by the Fisher’s exact test and the comparison of median age was performed using the Wilcoxon rank-sum test.
